# Temporal and regional progression of Alzheimer’s disease‐like pathology in 3xTg‐AD mice

**DOI:** 10.1111/acel.12873

**Published:** 2018-11-28

**Authors:** Ramona Belfiore, Alexis Rodin, Eric Ferreira, Ramon Velazquez, Caterina Branca, Antonella Caccamo, Salvatore Oddo

**Affiliations:** ^1^ The Arizona State University‐Banner Neurodegenerative Disease Research Center at the Biodesign Institute Arizona State University Tempe Arizona; ^2^ Department of Biomedical and Biotechnological Sciences University of Catania Catania Italy; ^3^ School of Life Sciences Arizona State University Tempe Arizona

**Keywords:** APP, Aβ, cognitive deficits, inflammation, microglia, neuroinflammation, plaques, tangles, tau, transgenic mice

## Abstract

Accumulation of amyloid‐β (Aβ) and fibrillary tangles, as well as neuroinflammation and memory loss, are hallmarks of Alzheimer’s disease (AD). After almost 15 years from their generation, 3xTg‐AD mice are still one of the most used transgenic models of AD. Converging evidence indicates that the phenotype of 3xTg‐AD mice has shifted over the years and contradicting reports about onset of pathology or cognitive deficits are apparent in the literature. Here, we assessed Aβ and tau load, neuroinflammation, and cognitive changes in 2‐, 6‐, 12‐, and 20‐month‐old female 3xTg‐AD and nontransgenic (NonTg) mice. We found that ~80% of the mice analyzed had Aβ plaques in the caudal hippocampus at 6 months of age, while 100% of them had Aβ plaques in the hippocampus at 12 months of age. Cortical Aβ plaques were first detected at 12 months of age, including in the entorhinal cortex. Phosphorylated Tau at Ser202/Thr205 and Ser422 was apparent in the hippocampus of 100% of 6‐month‐old mice, while only 50% of mice showed tau phosphorylation at Thr212/Ser214 at this age. Neuroinflammation was first evident in 6‐month‐old mice and increased as a function of age. These neuropathological changes were clearly associated with progressive cognitive decline, which was first apparent at 6 months of age and became significantly worse as the mice aged. These data indicate a consistent and predictable progression of the AD‐like pathology in female 3xTg‐AD mice, and will facilitate the design of future studies using these mice.

## 1 INTRODUCTION

Alzheimer’s disease (AD) is the most common neurodegenerative disease (Alzheimer’s Association, 2016). It is characterized by the accumulation of extracellular amyloid plaques and intracellular neurofibrillary tangles (LaFerla & Oddo, 2005; Querfurth & LaFerla, 2010). The former are primarily made of a small peptide called amyloid‐β (Aβ), while the latter are made of hyperphosphorylated tau (Querfurth & LaFerla, 2010). Another hallmark of AD is brain inflammation, which manifests as intense reactivity of astrocytes and microglia, and increased levels of proinflammatory cytokines such as interleukin‐1β (IL1β), interleukin‐6 (IL6), and tumor necrosis factor‐α (TNFα; Heneka et al., 2015). Clinically, AD is first associated with episodic amnesia followed by significant deficits in semantic memory and procedural memory (Mormino et al., 2009). Additional clinical manifestations such as loss of judgment, problem‐solving impairment, depression, and sleep disorders are also frequently associated with early stages of the disease (Grontvedt et al., 2018).

Familial AD, which represents a minority of AD cases, is due to mutations in one of three genes, presenilin (PS) 1 and 2 and the amyloid precursor protein (APP; Selkoe, 1997; Van Cauwenberghe, Broeckhoven, & Sleegers, 2016). In contrast, sporadic AD, which represents the vast majority of AD cases is of unknown etiology (Pluta, 2011; Zetterberg & Mattsson, 2014). For both familial and sporadic AD, aging is the major risk factor. Genetically, tau is not linked to AD, even though neurofibrillary tangles are a primary hallmark of AD. Mutations in the tau gene are associated with frontotemporal dementia, corticobasal degeneration, progressive supranuclear palsy, and Pick’s disease (Tacik, Sanchez‐Contreras, Rademakers, Dickson, & Wszolek, 2016).

Several animal models of AD have been generated and these include transgenic mice overexpressing different AD‐associated proteins (LaFerla & Green, 2012; Puzzo, Gulisano, Palmeri, & Arancio, 2015). Overall, mouse models of AD represent unique tools to understand underlying mechanisms of pathogenesis and have served to conduct numerous preclinical studies. In 2003, we generated a mouse model harboring three human mutant genes, APP, tau, and PS1 (Oddo, Caccamo, Kitazawa, Tseng, & LaFerla, 2003; Oddo, Caccamo, Shepherd, et al., 2003). These mice recapitulate specific aspects of AD including age‐dependent cognitive decline, accumulation of plaques and tangles, and age‐dependent inflammation (Janelsins et al., 2005; Oddo et al., 2008; Oddo, Caccamo, Kitazawa, et al., 2003; Oddo, Caccamo, Shepherd, et al., 2003). Over the last 15 years these mice have been widely used by investigators throughout the world. This widespread use of the mice has led to the generation of sublines, which present different onset and progression of AD‐like pathology, creating controversy and confusion in the field. Here we staged the AD‐like pathology in female 3xTg‐AD mice, to elucidate the spatial and temporal progression of soluble and insoluble Aβ, tau hyperphosphorylation, glial reactivity, and cognitive function.

## 2 RESULTS

### 2.1 3xTg‐AD mice show an age‐dependent accumulation of plaques

**Figure 1:**
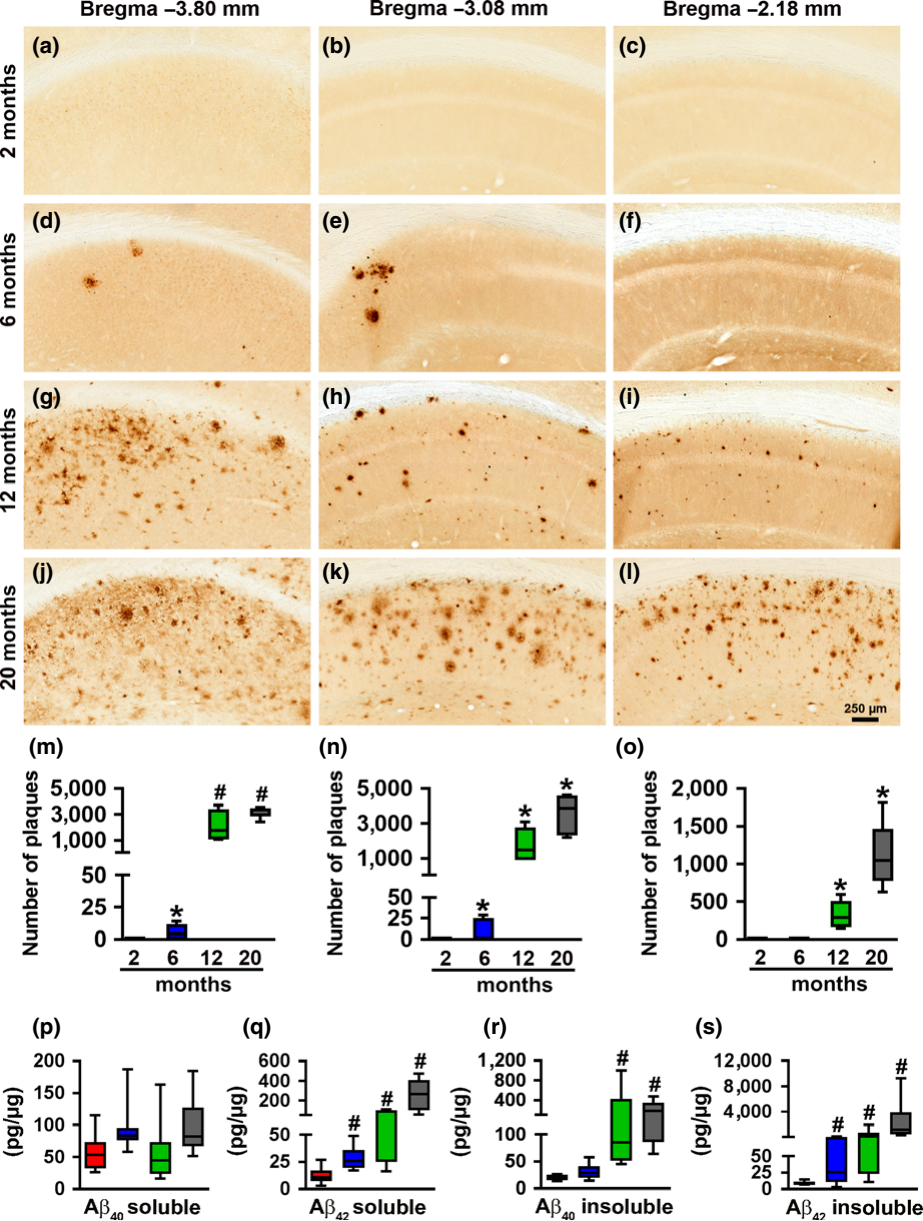
Age‐dependent Aβ pathology in the hippocampus. (a–l) Representative microphotographs of hippocampal sections from 2‐, 6‐, 12‐, and 20‐month‐old 3xTg‐AD mice stained with an anti‐Aβ_42_‐specific antibody (*n* = 6/age group). As indicated, brain sections were selected at −3.80, −3.08, and −2.18 mm posterior to bregma. (m–o), Quantitative analysis of the anti‐Aβ_42_ immunoreactivity from the caudal (*p* < 0.0001, *F*_3,20_ = 39.97), medial (*p* < 0.0001, *F*_3,20_ = 32.6) and rostral (*p* < 0.0001, *F*_3,20_ = 26.74) hippocampus. Post hoc analysis indicated that Aβ_42_ immunoreactivity in the caudal hippocampus (m) was significantly increased between 6 and 12 months of age (*p* < 0.0001) but not significantly increased between 12‐ and 20‐month‐old mice 3xTg‐AD (*p* = 0.0764). In contrast, a quantitative analysis of medial (n) and rostral (o) hippocampus showed significant differences among all the age‐groups (*p* < 0.001). (p–s), Sandwich ELISA measurements of soluble and insoluble Aβ_40 _and Aβ_42_ levels. The levels of soluble Aβ_40 _did not change as a function of age, whereas soluble Aβ_42 _levels were significantly different among the four age‐groups (*p* < 0.0001, *F*_3,31_ = 19.74). Specifically, post hoc evaluations showed that the levels of soluble Aβ_42 _were significantly different between all the pairwise comparisons (*p* < 0.0001) except when comparing the 6‐ and 12‐month‐old mice. Insoluble Aβ_40_ changed as a function of age (*p* = 0.0081, *F*_3,31_ = 4.699). Post hoc analyses indicated that the 12‐ and 20‐month groups were significantly different than the other two groups (*p* < 0.0001 for both comparisons). Insoluble Aβ_42_ levels also increased as a function of age (*p* < 0.0001, *F*_3,30_ = 15.15). Post hoc analyses showed the insoluble Aβ_42 _levels were significantly different between all the pairwise comparisons (*p* < 0.005), except when comparing the 6‐ and 12‐month‐old mice. Red bars indicate 2‐month‐old mice; blue bars indicate 6‐month‐old mice; green bars indicate 12‐month‐old mice; grey bars indicate 20‐month‐old mice. Data were analyzed by one‐way ANOVA followed by Bonferroni’s post hoc tests. Asterisks indicate differences within all the groups; hashtags indicate differences between selected groups. Error bars represent mean ± *SEM*

To assess the age‐dependent progression of Aβ pathology in female 3xTg‐AD mice, we immunostained sections from 2‐, 6‐, 12‐, and 20‐month‐old mice with an Aβ_42_‐specific antibody (*n* = 6/age group). We selected hippocampal sections from left hemibrains that were −3.80, −3.08, and −2.18 mm posterior to bregma, representing the caudal, medial, and rostral hippocampus, respectively. We found that extracellular Aβ plaques were absent in 2‐month‐old mice (Figure 1a–c) and first detected in the CA1/subiculum of 6‐month‐old mice (Figure 1d–f). At this age, five out of six mice analyzed had plaques in the caudal hippocampus, three out of six in the medial hippocampus and none in the rostral‐hippocampus. In contrast, 100% of the 12‐month‐old mice had plaques in all three hippocampal regions (Figure 1g–i). By 20 months of age, Aβ_42_ immunoreactivity was present throughout the hippocampus (Figure 1j–l). Quantitative analyses of the sections confirmed the age‐dependent progression of Aβ immunoreactivity in the caudal, medial, and rostral hippocampus (*p* < 0.0001; Figure 1m–o). To further assess the progression of Aβ pathology, we measured the amount of soluble and insoluble Aβ in the hippocampus of 3xTg‐AD mice by sandwich ELISA. One‐way ANOVA analysis showed that soluble Aβ_40_ levels were not statistically different among the four age groups (*p* = 0.0685; Figure 1p). In contrast, we found a statistically significant age‐dependent increase in insoluble Aβ_40_ (*p* = 0.0081; Figure 1r), and soluble and insoluble Aβ_42_ (*p* < 0.0001; Figure 1q,s).

**Figure 2:**
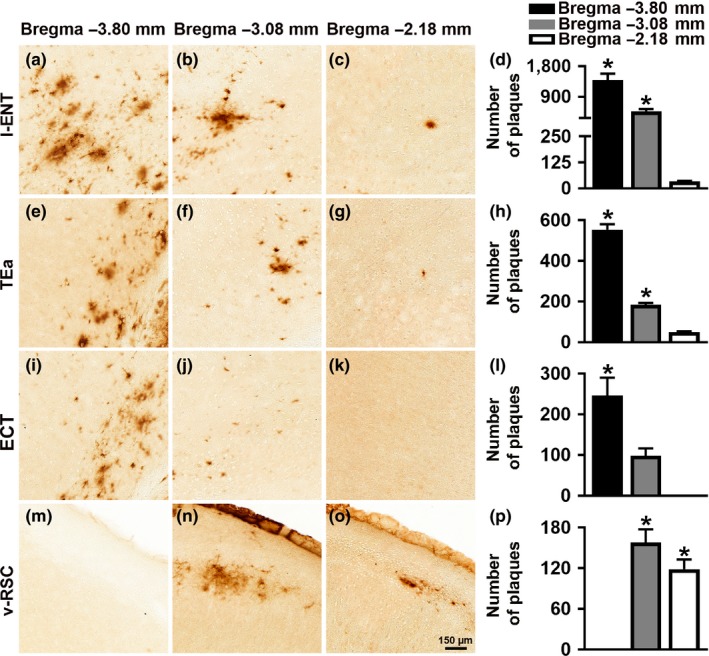
Cortical Aβ plaques deposition in 12‐month‐old 3xTg‐AD mice. Representative microphotographs of brain sections from 12‐month‐old 3xTg‐AD mice (*n* = 6/age group) stained with a selective Aβ_42_ antibody. Per each brain region, sections were taken at three different rostrocaudal levels. (a–c) Aβ_42_ immunoreactivity in the lateral entorhinal cortex (l‐ENT). (d) Quantitative analyses of the staining indicated that the number of Aβ plaques was higher in the caudal l‐ENT compared to the medial and rostral l‐ENT (*p* = 0.0013 and *p* < 0.0001, respectively). Also, there was a statistically significant difference between the medial and the rostral l‐ENT (*p* = 0.001). (e–g) Aβ_42_ immunoreactivity in the temporal association area (TEa). (h) Quantitative analyses of the staining indicated that in the caudal TEa the number of Aβ plaques was higher compared to medial and rostral TEa (*p* < 0.0001 for both comparisons). Further, the number of plaques was higher in the medial TEa compared to rostral TEa (*p* = 0.0008). (i–k) Aβ_42_ immunoreactivity in the ectorhinal cortex (ECT). (l) Quantitative analyses of the staining indicated that there was a higher number of Aβ plaques in the caudal ECT compared to medial and rostral ECT (*p* = 0.0125 and 0.0004, respectively). (m–o) Aβ_42_ immunoreactivity in the ventral retrosplenial cortex (v‐RSC). (p) Quantitative analyses of the staining indicated that the caudal v‐RSC had significantly fewer plaques compared to the medial and rostral v‐RSC (*p* = 0.003 and 0.004, respectively). Data were analyzed by one‐way ANOVA followed by Bonferroni’s post hoc evaluations. Asterisks indicate differences between all the groups. Error bars represent mean ± *SEM*

**Figure 3:**
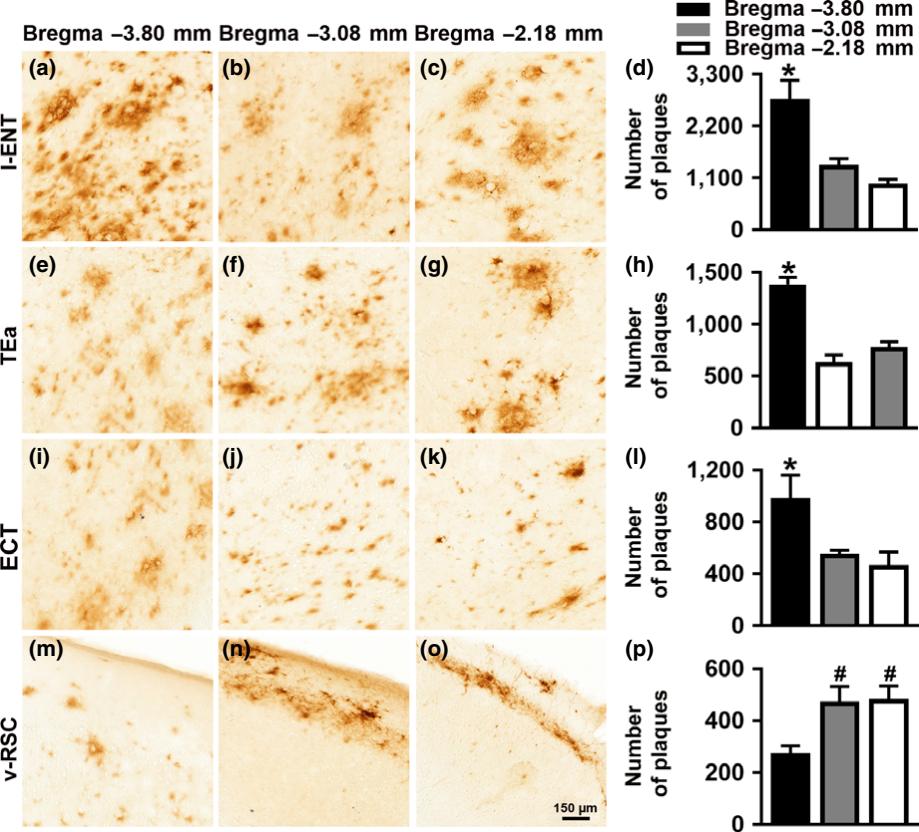
Cortical Aβ pathology in 20‐month‐old 3xTg‐AD mice. Representative microphotographs of brain sections from 20‐month‐old 3xTg‐AD mice (*n* = 6/age group) stained with a selective Aβ_42_ antibody. Per each brain region, sections were taken at three different rostrocaudal levels. (a–c) Aβ_42_ immunoreactivity in the lateral entorhinal cortex (l‐ENT). (d) Quantitative analyses of the staining indicated that the number of Aβ plaques was higher in the caudal l‐ENT compared to the medial and rostral l‐ENT (*p* = 0.0002 and *p* < 0.0001, respectively). (e–g) Aβ_42_ immunoreactivity in the temporal association area (TEa). (h) Quantitative analyses of the staining indicated that in the caudal TEa the number of Aβ plaques was higher than the medial and rostral TEa (*p* = 0.009 and 0.001, respectively). (i–k) Aβ_42_ immunoreactivity in the ectorhinal cortex (ECT). (l) Quantitative analyses of the staining indicated that there was a higher number of Aβ plaques in the caudal ECT compared to the medial and rostral ECT (*p* = 0.09 and 0.03, respectively. (m–o) Aβ_42_ immunoreactivity in the ventral retrosplenial cortex (v‐RSC). (p) Quantitative analyses of the staining indicated that the number of plaques was significantly increased in the medial and rostral v‐RSC compared to the caudal v‐RSC (*p* = 0.04 and 0.03, respectively). Data were analyzed by one‐way ANOVA followed by Bonferroni's post hoc evaluations. Asterisks indicate differences within all the groups; hashtags indicate differences with selected groups. Error bars represent mean ± *SEM*

To evaluate the cortical amyloid pathology, we measured Aβ load in the lateral entorhinal cortex (l‐ENT), temporal association area (TEa), ectorhinal cortex (ECT), and ventral retrosplenial cortex (v‐RSC). Aβ immunoreactivity was first apparent in the cortex of 12‐month‐old mice. Specifically, using one‐way ANOVA analysis, we found that at 12 months of age the number of Aβ plaques was higher in the caudal l‐ENT compared to both medial (*p* = 0.0013) and rostral l‐ENT (*p* < 0.0001; Figure 2a–d). We also observed this caudal‐rostral pattern for the TEa (*p* < 0.0001), and the ECT (*p* = 0.0004; Figure 2e–h and 2i–l, respectively). In contrast, we found that in the v‐RSC, Aβ_42_ immunoreactivity was significantly increased in the medial and rostral cortex compared to the caudal cortex (*p* < 0.0001 and *p* = 0.0006, respectively; Figure 2m–p). At 20 months of age, Aβ plaques were present throughout the cortex: a one‐way ANOVA analysis showed that the number of Aβ plaques was higher in the caudal compared to both medial and rostral l‐ENT (*p* = 0.0002 and *p* < 0.0001, respectively; Figure 3a–d), TEa (*p* = 0.009 and *p* = 0.001, respectively; Figure 3e–h), and ECT (*p* = 0.09 and *p* = 0.03, respectively; Figure 3i–l). In contrast, we found a higher number of Aβ plaques in the medial and rostral v‐RSC compared to the caudal v‐RSC (*p* = 0.04 and *p* = 0.03, respectively; Figure 3m–p). Together, these results indicate that in female 3xTg‐AD mice, Aβ plaques develop first in the caudal hippocampus and, as the mice age, they are present in the rostral hippocampus and several cortical regions.

### 2.2 3xTg‐AD mice show age‐dependent tau pathology

**Figure 4:**
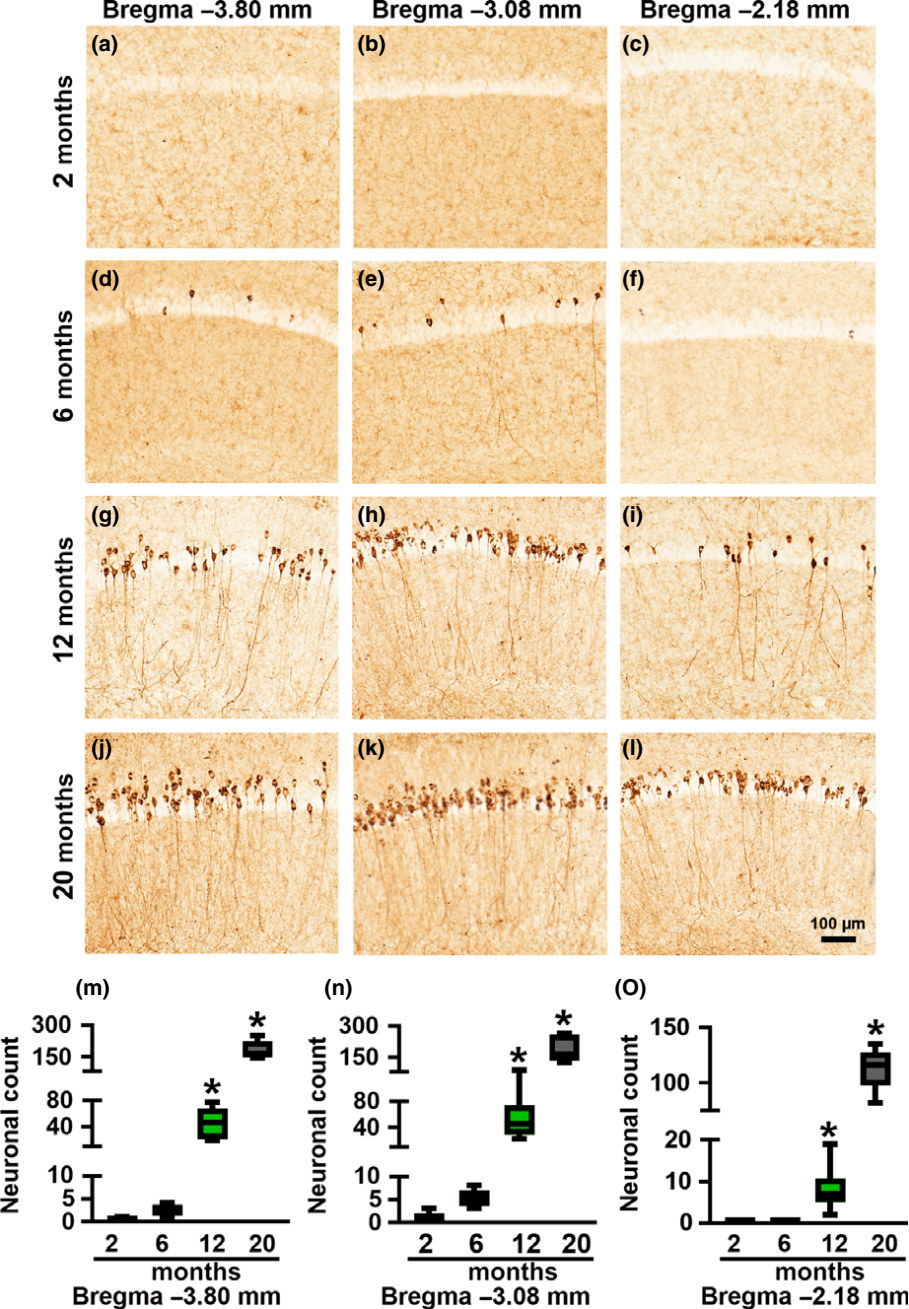
Age‐dependent progression of tau phosphorylation at S422 in hippocampi of 3xTg‐AD mice. (a–l) Representative microphotographs of hippocampal sections from 2‐, 6‐, 12‐, and 20‐month‐old 3xTg‐AD mice stained with an anti‐pS422‐specific antibody (*n* = 6/age group). Brain sections were selected at −3.80, −3.08, and −2.18 mm posterior to bregma. (m–o) Quantitative analysis of the anti‐pS422 immunoreactivity by one‐way ANOVA followed by a Bonferroni’s multiple‐comparison test shows a statistically significant age‐dependent difference in the caudal (*p* < 0.0001, *F*_3,20_ = 83.1), medial (*p* < 0.0001, *F*_3,20_ = 47.88) and rostral (*p* < 0.0001, *F*_3,20_ = 188.5) hippocampus. Post hoc analysis indicated that pS422 immunoreactivity in all three hippocampal regions was significantly higher in 12‐month‐old mice compared to the 2‐ and 6‐month groups (*p* < 0.005). Further, the 20‐month‐old mice have significantly higher pS422 immunoreactivity compared to all the other three age‐groups (*p* < 0.0001). Asterisks indicate differences within all the groups. Error bars represent mean ± *SEM*

To characterize the age‐related tau phosphorylation in female 3xTg‐AD mice, we immunostained sections from 2‐, 6‐, 12‐, and 20‐month‐old mice (*n* = 6/age group) with antibodies that recognize tau phosphorylated at Ser422 (pS422), Ser202/Thr205 (AT8), and Thr212/Ser214 (AT100). We found that tau pS422 immunoreactivity, an early marker in the progression of tau pathology in AD (Kanaan et al., 2016; Simic et al., 2016), was practically absent in 2‐month‐old mice (Figure 4a–c). In contrast, 100% of the 6‐month‐old mice analyzed showed pS422 immunoreactivity in the caudal and medial hippocampus but not in the rostral hippocampus (Figure 4d–f and Table 1). Finally, 100% of 12‐ and 20‐month‐old mice had extensive pS422‐positive neurons in all three brain regions (Figure 4g–l and Table 1). Overall, one‐way ANOVA indicated that in all three brain regions there was a significant age‐dependent increase in pS422 immunoreactivity (*p* < 0.0001; Figure 4m–o). A similar pattern in tau phosphorylation was detected with AT8 and AT100. To this end, we found no AT8 or AT100 immunoreactivity in 2‐month‐old mice (Supporting Information Figures S1 and S2). In contrast, AT8 immunoreactivity was present in 100% of the 6‐month‐old mice analyzed (Supporting Information Figure S1D–F), while AT100 immunoreactivity was present in 50% of 6‐month‐old mice (Figure S2D–F; Table 1). 100% of 12‐ and 20‐month‐old mice had extensive AT8 and AT100 immunoreactivity throughout the hippocampus (Supporting Information Figures S1G–L and S2G–L, respectively). One‐way ANOVA indicated that in all three hippocampal regions, AT8 and AT100 immunoreactivity significantly increased as a function of age (*p* < 0.0001 for both markers in all three hippocampal regions; Supporting Information Figures S1M–O and S2M–O).

**Table 1:** Age‐dependent progression of tau phosphorylation in 3xTg‐AD mice (*n* = 6/age group). The neuronal count is represented by mean ± *SEM*

Age	−3.80 mm		−3.08 mm		−2.18 mm	
	Positive neurons	% Mice	Positive neurons	% Mice	Positive neurons	% Mice
S422						
2 month	0.2 ± 0.20	33.3	1 ± 0.5400	50	0	0
6 month	5.2 ± 0.86	100	2.5 ± 0.64	100	0.3 ± 0.21	33
12 month	44.6 ± 11.01	100	50.2 ± 11.83	100	8.1 ± 2.33	100
20 month	182 ± 19.55	100	191 ± 28.85	100	113 ± 8.66	100
AT8						
2 month	0.3 ± 0.21	33.3	0	0	0	0
6 month	10 ± 1.59	100	2.2 ± 0.2	100	0.3 ± 0.21	33.3
12 month	44.6 ± 18.24	100	56.83 ± 11.04	100	21.83 ± 9.96	100
20 month	182 ± 12.89	100	187 ± 20.61	100	95.17 ± 18.25	100
AT100						
2 month	0	0	0	0	0	0
6 month	1.5 ± 0.64	50	2.2 ± 0.91	50	0	0
12 month	21.5 ± 3.83	100	55.6 ± 6.60	100	25.2 ± 12.96	100
20 month	107.5 ± 6.58	100	252.75 ± 34.6	100	85 ± 11.98	100

To evaluate cortical tau phosphorylation, we measured pS422, AT8 and AT100 immunoreactivity in the lateral entorhinal cortex (l‐ENT), temporal association area (TEa), ectorhinal cortex (ECT), and ventral retrosplenial cortex (v‐RSC). We first detected cortical tau pS422 immunoreactivity in 12‐month‐old mice. At this age, 100% of mice had a few pS422‐positive neurons in the medial l‐ENT cortex and 67% of mice in caudal and rostral l‐ENT cortex (Supporting Information Figure S3A–C). Indeed, using one‐way ANOVA analysis with Bonferroni’s correction, we found that at 12 months of age pS422 immunoreactivity was higher in the medial l‐ENT compared to both caudal and rostral l‐ENT (*p* < 0.0001 and *p* = 0.0261, respectively; Supporting Information Figure S3D). In contrast, pS422 immunoreactivity was not detected in the TEa, ECT, and v‐RSC of 12‐month‐old mice (Supporting Information Figure S3E–M). Notably, pS422 immunoreactivity increased as a function of age and 100% of 20‐month‐old mice had pS422‐positive neurons in both l‐ENT and TEa (Supporting Information Figure S4A–H). A one‐way ANOVA with Bonferroni’s correction showed that the number of pS422 positive neurons was higher in the medial cortex compared to both caudal and rostral l‐ENT cortex (*p* = 0.0006 and *p* = 0.0048, respectively; Supporting Information Figure S4D). In contrast, we found that pS422 immunoreactivity was not significantly different among the three bregma regions analyzed (Supporting Information Figure S4H). We also found no positive neurons for pS422 immunostaining in the ECT and v‐RSC.

We found a similar pattern in cortical tau phosphorylation with AT8 and AT100 antibodies. Specifically, cortical AT8 immunoreactivity was first detected at 12 months of age in the l‐ENT (Supporting Information Figure S5A–D), but it was not present in the other three cortical regions analyzed (Supporting Information Figure S5E–M). At this age, 100% of the mice examined had AT8‐positive neurons in the medial l‐ENT. Quantitative analyses indicated that within the l‐ENT, the number of AT8‐positive neurons was significantly higher in the medial l‐ENT compared to both caudal and rostral l‐ENT (*p* < 0.0001 and *p* = 0.0061, respectively; Supporting Information Figure S5D). In 20‐month‐old mice, AT8 immunoreactivity was more prominent in the l‐ENT and was present in 100% of the mice in all three regions of the l‐ENT (Supporting Information Figure S6A–D). At this age, AT8 immunoreactivity was very limited and a few AT8‐positive neurons were apparent in the caudal and medial TEa (Supporting Information Figure S6E–H). Quantitative analyses showed that the number of AT8‐positive neurons was higher in the medial l‐ENT compared to both caudal and rostral l‐ENT cortex (*p* < 0.0001; Supporting Information Figure S6D). In contrast, there were more AT8‐positive neurons in the caudal and medial TEa than the rostral TEa of 20‐month‐old mice (*p* < 0.0001, Supporting Information Figure S6H). We did not detect any AT8 immunoreactivity in the ECT and v‐RSC at this age (Supporting Information Figure S6I–N). Similarly, in 12‐month‐old mice a few AT100‐positive neurons were detected in the medial and rostral l‐ENT but not in the caudal l‐ENT (Supporting Information Figure S7A–D; *p* < 0.0001) or the other cortical regions analyzed (Supporting Information Figure S7E–M). In 20‐month‐old mice, we found prominent AT100 immunoreactivity in all three regions of the l‐ENT (Supporting Information Figure S8A–C). Statistical evaluation revealed that the number of AT100‐positive neurons was significantly higher in the medial and rostral l‐ENT compared to the caudal l‐ENT (*p* < 0.0001; Supporting Information Figure S8D). We did not detect AT100 immunoreactivity in the other three cortical regions analyzed of 20‐month‐old mice (Supporting Information Figure S8E–M). Together these results indicate that tau phosphorylation in the hippocampus first appears at 6 months of age, while in the I‐ENT is first detected at 12 months of age.

### 2.3 Astrogliosis and microgliosis in 3xTg‐AD mice

Inflammation is another hallmark of AD pathogenesis and is considered a sign of neuronal damage (Sudduth, Schmitt, Nelson, & Wilcock, 2013). To study the age‐dependent inflammatory events in female 3xTg‐AD mice, we first assessed astrogliosis in 2‐, 6‐, 12‐, and 20‐month‐old mice using a GFAP antibody (*n* = 6/age group). Compared to age‐ and sex‐matched NonTg mice, astrogliosis was increased in 12‐ and 20‐month‐old 3xTg‐AD mice in the caudal hippocampus (*p* = 0.0001 and *p* < 0.0001, for fluorescent intensity respectively, and *p* < 0.0001 and *p* < 0.0001 for number of cells, respectively; Supporting Information Figure S9A–B); in the medial hippocampus (*p* = 0.0054 and *p* = 0.0016, for fluorescent intensity respectively and *p* = 0.0001 and *p* < 0.0001 for number of cells, respectively; Supporting Information Figure S9C–D) and rostral hippocampus (*p* = 0.0255 and *p* = 0.0012 for fluorescent intensity, respectively and and *p* = 0.0004 and *p* < 0.0001 for number of cells, respectively; Supporting Information Figure S9E,F).

**Figure 5:**
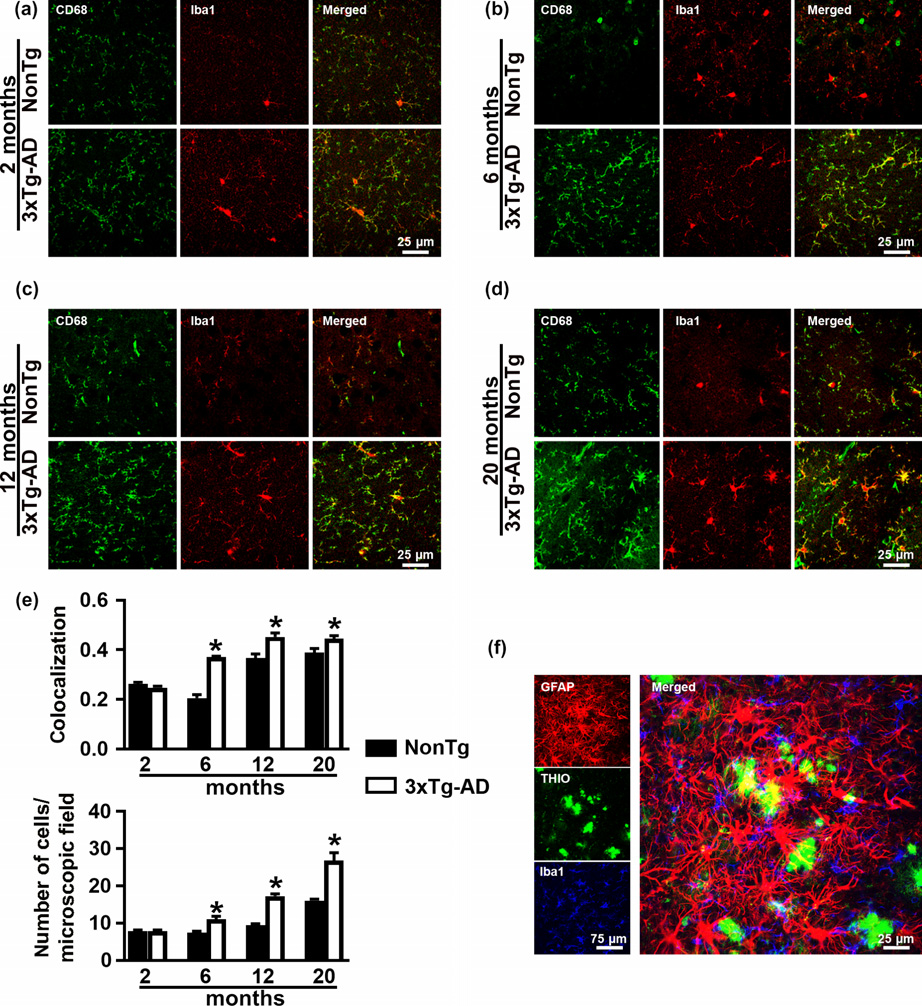
Age‐dependent microglia activation in 3xTg‐AD mice. (a–d) Representative confocal microphotographs of CA1 sections from the medial hippocampus of NonTg and 3xTg‐AD mice (*n = *6/genotype/age group). Sections were stained antibodies against Iba1 and CD68. (e) Quantitative analysis revealed that the number of colocalized pixels (top graph) and the number of Iba1‐positive cells (bottom graph) was significantly higher in 3xTg‐AD mice than NonTg mice at 6, 12 and 20 months. (f) Representative confocal microphotographs of CA1 section from 20‐month‐old 3xTg‐AD mice stained with Thioflavin (green), anti‐GFAP (red), and anti‐Iba1 (blue) antibodies. The image shows the different distribution of microglial (homogenously distributed) and reactive astrocytes (surrounding Aβ plaques). Statistical evaluation was obtained by the Pearson’s correlation coefficients. Error bars represent mean ± *SEM*

To evaluate microglial activation, we co‐labeled sections from 2‐, 6‐, 12‐, and 20‐month‐old 3xTg‐AD and NonTg mice with Iba1 and CD68 antibodies (*n* = 6/age group). The former recognizes total microglia, while the latter is a lysosomal marker. Notably, CD68 levels are lower in quiescent microglia and extensively increased in highly reactive microglia (Zotova et al., 2011). Therefore, colocalization of CD68 and Iba1 signals indicates activated microglia. At 2 months of age, there was no difference in microglia activation between 3xTg‐AD and NonTg mice in the medial hippocampus as indicated by the intensity of the yellow pixels and the number of CD68 positive cells (Figure 5a,e). In contrast, starting at 6 months of age, there was a significant age‐dependent increase in microglia activation in the medial hippocampus of 3xTg‐AD mice compared to age‐matched NonTg mice (*p* < 0.0001 for fluorescent intensity and *p* < 0.01 for number of Iba1‐positive cells; Figure 5b–e). A similar pattern was evident in the caudal and rostral hippocampus. (Supporting Information Figure S10A–E, Figure S11A–E, respectively). An observational analysis of triple labeling with Thioflavin S, GFAP and Iba1, showed that astrocytes were present specifically around Aβ plaques while reactive microglia were homogeneously detected along the CA1 of 3xTg‐AD mice hippocampi (Figure 5f). These results indicate that in female 3xTg‐AD mice microglia activation, first detected at 6 months of age, precedes astrogliosis, first detected at 12 months of age.

To determine whether the age‐dependent neuropathological changes observed in 3xTg‐AD mice were linked to changes in transgene expression, we measured the steady‐state levels of human APP (with 6E10) and human tau (with HT7). We found that APP and tau levels were similar across the four age‐groups as indicated by one‐way ANOVA (*p* = 0.48 and 0.42 for APP and tau, respectively; Supporting Information Figure S12).

### 2.4 Learning and memory impairments in 3xTg‐AD mice

**Figure 6:**
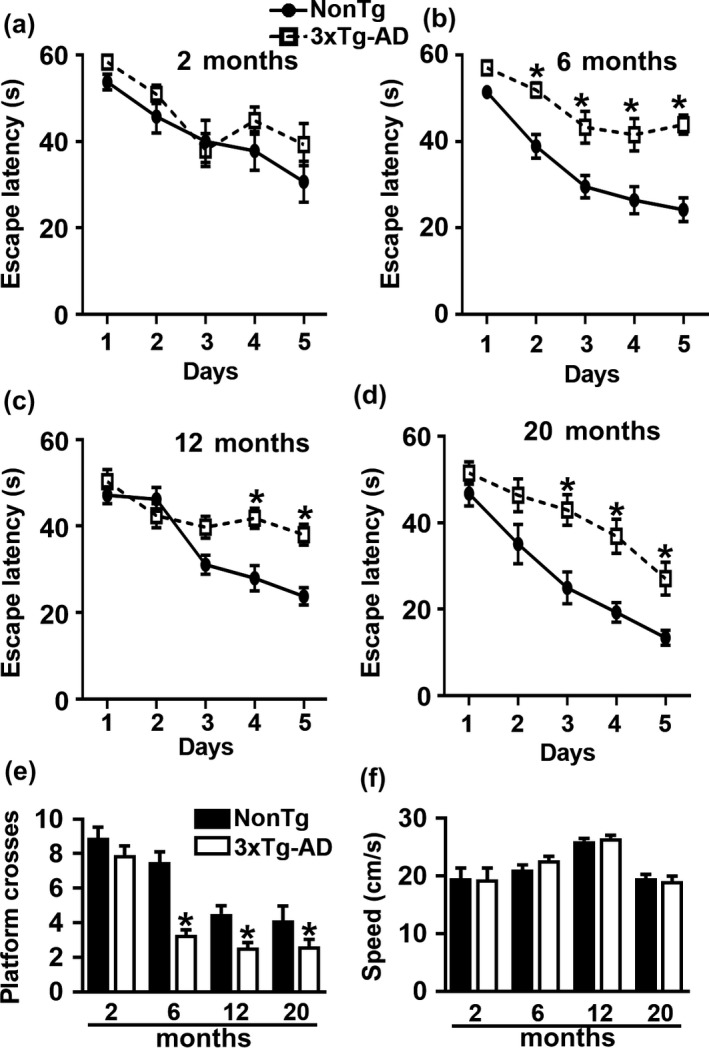
Age‐dependent spatial learning and memory deficits. (a–d) Learning curves of mice trained in the spatial reference version of the Morris water maze (2 months, *n* = 15/genotype; 6 months, *n* = 15 for 3xTg‐AD and *n* = 10 for NonTg; 12 months, *n* = 15 for 3xTg‐AD and *n* = 14 for NonTg; and 20 months, *n* = 15 for 3xTg‐AD and *n* = 11 for NonTg). The escape latency to find the hidden platform was plotted against the days of training. The values for each day represent the average of four training trials. At 2 months of age, we found a significant effect for day (*p < *0.0001, *F*_4,135_ = 10.90) but not a significant effect for genotype (*p* = 0.0623, *F*_1,135_ = 3.53). At 6 months of age, we found significant effects for day (*p < *0.0001, *F*_4,92_ = 23.37) and genotype (*p < *0.0001, *F*_1,23_ = 41.86). Post hoc tests indicated that NonTg mice performed significantly better than 3xTg‐AD mice on day 2 (*p* = 0.0053), day 3 (*p* = 0.0028), day 4 (*p* = 0.0008), and day 5 (*p* < 0.0001). At 12 months of age, there was a significant effect for day (*p < *0.0001, *F*_4,108_ = 21.85) and genotype (*p = *0.0022, *F*_1,27_ = 11.46). Post hoc tests indicated that NonTg mice performed significantly better than 3xTg‐AD mice on day 4 (*p* = 0.0008) and day 5 (*p* = 0.0005). At 20 months of age, there was a significant effect for day (*p < *0.0001, *F*_4,92_ = 26.03) and for genotype (*p < *0.0001, *F*_1,23_ = 22.05). Post hoc tests showed that 3xTg‐AD mice performed significantly worse when compared with NonTg mice on day 3 (*p* = 0.0014), day 4 (*p* = 0.0018) and day 5 (*p* = 0.0259). (e) Number of platform location crosses during a single 60‐s probe trial. 3xTg‐AD mice performed significantly worse compared to NonTg mice at 6 (*p* = 0.0008), 12 (*p* = 0.0123), and 20 months of age (*p* = 0.01400). (f) Swim speed was similar between the two groups at all the ages (*p* > 0.05). Learning data were analyzed by two‐way ANOVA; probe trials were analyzed by one‐way ANOVA. Bonferroni’s was used for post hoc tests. *Significant difference between NonTg and 3xTg‐AD mice. Error bars represent mean ± *SEM*

To assess spatial learning and memory, we tested 2‐, 6‐, 12‐, and 20‐month‐old female 3xTg‐AD and NonTg mice on the spatial version of the Morris water maze (2 months, *n* = 15/genotype; 6 months, *n* = 15 for 3xTg‐AD and *n* = 10 for NonTg; 12 months, *n* = 15 for 3xTg‐AD and *n* = 14 for NonTg; and 20 months, *n* = 15 for 3xTg‐AD and *n* = 11 for NonTg). We gave mice four training trials per day for five consecutive days to learn the location of a hidden platform using distal extra‐maze cues. The escape latency to find the platform across the training trials is an indication of mouse learning, with less time interpreted as better learning. Using a mixed ANOVA, we found that at 2 months of age there was a significant effect for days (*p* < 0.0001) and no significant effect per genotype (*p* = 0.0699) or genotype × day interaction (*p* = 0.681; Figure 6a). These results suggest that mice learn the task across days but there is no difference in the pace of learning between the two genotypes. In contrast, we found that at 6 months of age there was a significant effect for days (*p* < 0.0001), genotype (*p* < 0.0001), and a significant genotype × day interaction (*p* = 0.0478; Figure 6b). Notably, a post hoc test with Bonferroni’s correction showed that NonTg mice performed significantly better than 3xTg‐AD mice on day 2 (*p* = 0.0053), day 3 (*p* = 0.0028), day 4 (*p* = 0.0008) and day 5 (*p* < 0.0001). As the mice aged, the difference in learning became more pronounced. To this end, we found that at 12 months of age, there was a significant effect for days (*p* < 0.0001), genotype (*p* = 0.0022) and genotype × day interaction (*p* = 0.0003; Figure 6c). A post hoc test with Bonferroni’s correction showed that 3xTg‐AD mice were significantly impaired on day 4 and day 5 compared with NonTg mice (*p* = 0.0008; *p* = 0.0005, respectively; Figure 6c). Finally, at 20 months of age, there was a significantly different effect for days (*p* < 0.0001), genotype (*p* = 0.0022) and genotype × day interaction (*p* = 0.0003; Figure 6d) in the escape latency. Notably, post hoc analyses with Bonferroni's correction showed that NonTg mice performed better than 3xTg‐AD mice on day 3, 4, and 5 (*p* = 0.0014, *p* = 0.0018 and *p* = 0.0259, respectively; Figure 6d).

Twenty‐four hours after the last training trial, we conducted probe trials to measure spatial reference memory. Specifically, we measured the number of platform location crosses during a single 60‐s trial. We found that 3xTg‐AD mice performed significantly worse when compared to the NonTg at 6, 12, and 20 months of age (*p* = 0.0008, *p* = 0.0123, *p* = 0.01400, respectively; Figure 6e). We found that the swim speed was not statistically significant among the two genotypes (Figure 6f), indicating that the genotype effects on learning and memory were independent of physical performance at all ages. These findings indicate a clear age‐dependent cognitive decline in 3xTg‐AD mice.

## 3 DISCUSSION

Animal models are invaluable tools to study mechanisms of AD pathogenesis. Most models have been generated using human mutations in the APP or PS1 genes that are associated with familial AD (LaFerla & Green, 2012). However, these mice fail to recapitulate the full spectrum of AD pathology, despite developing a high degree of Aβ plaques. In contrast, overexpression of wild‐type tau does not lead to any phenotype, while expression of a human mutant tau leads to a strong tau pathology, often associated with neurodegeneration (LaFerla & Green, 2012; Puzzo et al., 2015). In 2013, we generated the 3xTg‐AD mice, which harbor mutations in APP, PS1, and tau genes. These mice develop Aβ and tau pathology, as well as neuroinflammation and cognitive deficits (Billings, Oddo, Green, McGaugh, & LaFerla, 2005; Kitazawa, Oddo, Yamasaki, Green, & LaFerla, 2005; Oddo, Caccamo, et al., 2006; Oddo, Caccamo, Kitazawa, et al., 2003; Oddo, Caccamo, Shepherd, et al., 2003; Oddo, Vasilevko, et al., 2006). While 3xTg‐AD mice overexpress mutant tau, which is not associated with AD, they have been invaluable in understanding the interplay between Aβ and tau. Multiple reports have consistently shown that in these mice, Aβ pathology contributes to the development of tau (Oddo et al., 2007, 2008; Oddo, Billings, Kesslak, Cribbs, & LaFerla, 2004; Oddo, Caccamo, Cheng, & LaFerla, 2009; Oddo, Caccamo, et al., 2006; Oddo, Vasilevko, et al., 2006). Further, we previously showed that removing Aβ was sufficient to improve tau pathology (Oddo et al., 2004; Oddo, Caccamo, et al., 2006; Oddo, Vasilevko, et al., 2006). Similar results have been obtained from human clinical trials in which Aβ immunization led to the clearance of tau (Amin et al., 2015; Boche et al., 2010; Gilman et al., 2005). Together, these results highlight how findings in 3xTg‐AD mice in regards to Aβ and tau interaction have predicted results in humans.

Our data indicate that at early ages there is a rostral‐caudal gradient of Aβ and tau pathology in the hippocampus. Indeed, we found that Aβ plaques and tau phosphorylation were first apparent in the caudal hippocampus of 6‐month‐old mice; however, at the same age, none of the mice analyzed had Aβ plaques or evidence of tau phosphorylation in more rostral areas of the hippocampus. Consistent with our observations, MRI studies of 120 participants of the AD Neuroimaging Initiative showed that hippocampal subregions undergo differential atrophy in AD (Greene, Killiany, & Alzheimer’s Disease Neuroimaging Initiative, 2012). Functionally, the rostral and caudal hippocampus appeared to be involved in different forms of learning and memory. To this end, functional MRI studies have clearly indicated two subnetworks within the medial temporal lobe memory system, one involving the rostral hippocampus, and one involving the medial hippocampus (Greene et al., 2012; Kahn, Andrews‐Hanna, Vincent, Snyder, & Buckner, 2008; Ranganath & Ritchey, 2012). The integrity of these networks is differentially regulated during aging and AD (Dickerson, Brickhouse, McGinnis, & Wolk, 2017). While the significance behind these observations remains to be elucidated, it is tempting to speculate that the rostral hippocampus, at least at early ages, might be more resilient to the development of AD‐like pathology.

Phosphorylation of tau at Ser422 is considered a disease‐specific event and is associated with tau misfolding in AD and other tauopathies (Bussiere et al., 1999; Guillozet‐Bongaarts et al., 2006). We found that Ser422 is one of the earliest tau amino acids to be phosphorylated in 3xTg‐AD mice and that Ser422 immunoreactivity increases as a function of age. Consistent with our observations, tau phosphorylation at Ser422 occurs early in the disease and remains phosphorylated through the disease progression (Augustinack, Schneider, Mandelkow, & Hyman, 2002). As the 3xTg‐AD mice age, tau becomes more phosphorylated at other epitopes (Mastrangelo & Bowers, 2008; Oddo et al., 2009). Here we show that AT8 and AT100 immunoreactivity occurs at middle and late stages of the disease progression, respectively. These findings are consistent with what has been reported in other transgenic mice expressing mutant human tau (Augustinack et al., 2002) and postmortem human AD brains. Along these lines, a large body of literature indicates that in people the accumulation of plaques and tangle is not always associated with cognitive decline as plaque or tangles deposition is often found in cognitive normal people. In contrast, it has been suggested that soluble, more toxic forms of Aβ and tau might be associated with cognitive deficits. This hypothesis also applies to 3xTg‐AD mice. Indeed, at the onset of cognitive deficits, while the mice are deprived of plaques and tangles, they already have high levels of soluble Aβ_42_ and accumulation of phosphorylated tau.

Overall these results indicate that there is a slight temporal delay in the onset of pathology in 3xTg‐AD mice with what we and others have reported in the past (Mastrangelo & Bowers, 2008). Nevertheless, the phenotype of female 3xTg‐AD mice is consistent, predictable, and shows a close association with that observed in AD patients and other animal models (Bilkei‐Gorzo, 2014; Braak, Alafuzoff, Arzberger, Kretzschmar, & Tredici, 2006; Duyckaerts, Potier, & Delatour, 2008). While the presence of the mutant tau gene does not reflect the condition in AD, these mice continue to be an invaluable tool to study the interaction between Aβ and tau. As discussed above, many findings were predictive to what was later observed in human brains. Further, 3xTg‐AD mice show a clear interaction between age and development of the phenotype, which makes them an excellent tool to study the role of aging in the disease pathogenesis.

## 4 EXPERIMENTAL PROCEDURES

### 4.1 Animals

The 3xTg‐AD mice used in this manuscript were previously described (Oddo, Caccamo, Kitazawa, et al., 2003; Oddo, Caccamo, Shepherd, et al., 2003). They were obtained from the original colony generated at the University of California, Irvine in 2001 and published in 2003. The 3xTg‐AD mice were generated on a C57BL6/129SvJ hybrid background (Oddo, Caccamo, Kitazawa, et al., 2003; Oddo, Caccamo, Shepherd, et al., 2003). Given that the mice are homozygous for the mutations in the PS1, APP, and tau genes, we maintained the colony by breeding homozygous 3xTg‐AD mice to each other. In this study, only female mice were analyzed since in our colony male 3xTg‐AD mice show a sizeable neuropathological variability, even between littermates. Mice were housed 4–5 per cage, kept on 12‐hr light/dark cycle, and were given ad libitum access to food and water. All animal procedures were approved by The Institutional Animal Care and Use Committee of Arizona State University.

### 4.2 Protein extraction

Brains from NonTg and 3xTg‐AD mice were processed for biochemical analyses as described previously (Caccamo, Belfiore, & Oddo, 2018). Briefly, mice were killed by CO_2 _asphyxiation, their brain removed and sagittally bisected. The left hemispheres were used for immunohistochemical analysis; the right hemispheres were dissected to separate hippocampus, cortex, and cerebellum and frozen in dry ice. The hippocampal tissue was homogenized in T‐PER protein extraction buffer (Thermo Fisher, Waltham, MA), containing complete protease inhibitor (Roche, Indianapolis, IN) and phosphatase inhibitor (Thermo Fisher). The homogenates were centrifuged at 4°C for 30 min at 100,000 *g*. The supernatant containing the soluble protein fraction was stored at −80°C and used for ELISA and western blots. The insoluble fraction was obtained resuspending the pellet in 70% formic acid and used to measure insoluble Aβ by ELISA.

### 4.3 ELISA

To detect soluble and insoluble Aβ_40_ and Aβ_42_, we used a commercially available sandwich ELISA kit from Thermo Fisher Scientific as previously described (Velazquez, Shaw, Caccamo, & Oddo, 2016). Briefly, soluble or insoluble fractions of brain tissue homogenates were processed in a precoated, flat bottom 96‐well plates according to the kit’s instructions and read in a BioTek plate reader at 450 nm. The range of Aβ detection was between 10 and 1,000 pg/ml. For each assay kit, cross‐reactivity with other Aβ species was negligible when concentrations were <10 ng/ml. The concentration of Aβ (picograms per milliliter of sample) present in the homogenate was the dependent variable used for statistical analysis.

### 4.4 Morris water maze

Morris water maze tests were conducted as previously described (Caccamo et al., 2017). Briefly, mice were tested in a circular tank of 1.5 m in diameter located in a room with extra‐maze cues. The platform (14 cm in diameter) location was maintained constant for each mouse during training and was submerged 1.5 cm beneath the surface of the water, which was maintained at 23°C throughout the testing. During 5 days of training, the mice underwent four trials a day, alternating among four pseudorandom starting points. If a mouse failed to find the platform within 60 s, it was guided to the platform by the researcher and kept there for 10 s. The inter‐trial interval was 25 s, during which time each mouse was returned to its home cage. Probe trials were conducted 24 hr after the last training trial. During the probe trials, the platform was removed and mice were free to swim in the tank for a single 60‐s trial. The training and probe trials were recorded by a video camera and were analyzed using the EthoVisio XT tracking system (Noldus Information Technology, Leesburg, VA). The outcome of the Morris water maze is very sensitive to ambient changes during the testing, including changes in the air pressure in the room, the water temperature, and background noise. To counteract these possible confounds, for each age, we ran all the 3xTg‐AD and NonTg mice at the same time. Doing so, we can compare the results of the two genotypes to each other. However, mice of different ages were run at different times and thus, the results should not be compared across the different ages.

### 4.5 Antibodies

From Millipore, Aβ_42_ (catalog number AB5078P, 1:200). From Thermo Fisher Scientific, p‐tau Thr212/Ser214 (AT100; catalog number MN1060, 1:1,000); p‐tau Ser202/Thr205 (AT8; catalog number MN1020, 1:1,000); Goat anti‐Rabbit IgG (H + L) highly cross‐adsorbed secondary antibody; Alexa Fluor Plus 555 and 488 (catalog number A32732 and A‐11034, respectively; 1:200); Goat anti‐Mouse IgG (H + L) highly cross‐adsorbed secondary antibody; and Alexa Fluor Plus 488 (catalog number A32723; 1:200). From Vector Labs, biotinylated goat anti‐rabbit and mouse IgG antibody (catalog number BA‐1000 and BA‐2001; 1:200). From Wako, Iba1 (catalog number 09‐19741, 1:500). From Cell Signaling, GFAP (catalog number 3670S, 1:500) and β‐actin (catalog number 3700, 1:10,000). From GenTex, tau pSer422 (catalog number GTX86147, 1:200). From Abcam, CD68 (catalog number ab955, 1:500).

### 4.6 Immunohistochemistry and immunofluorescence

Brains were processed as previously described (Caccamo et al., 2017). Briefly, hemibrains were drop fixed in 4% paraformaldehyde for 48 hr and then transferred into 0.02% sodium azide in phosphate‐buffered saline until slicing; 50‐μm‐thick free‐floating sections were subsequently obtained using a vibratome. For immunohistochemistry, sections were washed twice with TBS (100 mm Tris pH 7.4, 150 mm NaCl) and incubated for 30 min in 3% H_2_O_2_, to quench endogenous peroxidase activity. Next, sections were transferred into TBS‐A (100 mm Tris pH 7.4, 150 mm NaCl, 0.1% Triton X‐100) and TBS‐B (100 mm Tris pH 7.4, 150 mm NaCl, 0.1% Triton X‐100, 2% bovine serum albumin) for 15 and 30 min, respectively. Finally, the proper primary antibody was applied overnight at 4°C. Sections were washed to remove excess antibody and incubated in the suitable secondary antibody for one h at room temperature. Signal was enhanced by incubating sections in the avidin‐biotin complex (Vector Labs, Burlingame, CA, USA) for one h. Sections were then washed and developed with diaminobenzidine substrate using the avidin‐biotin horseradish peroxidase system (Vector Labs). Images were obtained with a digital Zeiss camera and analyzed using ImageJ. For immunofluorescence staining, the quenching step was skipped, and after the secondary antibody (AlexaFluor; Thermo Fisher Scientific), the slices were mounted and coverslip with Prolong^®^ diamond mounting (Thermo Fisher Scientific). For all colocalization measurements, lasers 561 and 488 nm were used for excitation of secondary antibody fluorophores Alexa 555 and Alexa 488, respectively (Thermo Fisher Scientific). To obtain a Pearson correlation coefficient (PCC), we used ImageJ plugin “Coloc2.” To quantify Aβ and tau immunoreactivity, images from six mice/group were taken with a Zeiss AxioImager A1 using a 40× objective. To quantify colocalization in confocal imaging, we quantified and averaged 10 pictures per mouse, six mice per genotype. To count astrocytes and microglia, GFAP‐ and Iba1‐ stained sections were visualized using a 40× objective with a 1.5× digital zoom. Per each sections, we randomly took six pictures of the hippocampus and analyzed a total of six mice per genotype. Pictures were then transferred to ImageJ for cell counting. Images were analyzed using ImageJ.

### 4.7 Western blot

Western blots were performed under reducing conditions using precast Novex gels from Life Technologies. Proteins were transferred to nitrocellulose membranes with iBlot (Life Technologies), and incubated for 60 min in 5% nonfat milk (Great Value) in Tris‐buffered saline with Tween (TBST; 0.1 m Tris, 0.15 m NaCl, and 0.1% Tween 20). Primary antibodies specific to the experiment were applied overnight at 4°C in 5% milk in TBST buffer. The next day, blots were washed in TBST three times for 10 min and then incubated in the appropriate fluorescent secondary antibody for 1 hr, at room temperature. The blots were then washed as described above, and imaged were quantified using a LI‐COR Odyssey CLx (LI‐COR Biosciences) attached to a Dell computer (OptiPlex 7010) running Windows 7 and Image Studio (version 1.0.11, LI‐COR Biosciences).

### 4.8 Statistical analysis

Data were analyzed by Student’s *t* test, one‐way and two‐way ANOVAs using GraphPad Prism. Post hoc with Bonferroni’s correction was used when appropriate.

## CONFLICT OF INTERESTS

The authors have no conflict of interest.

## AUTHOR CONTRIBUTIONS

RB performed most of the experiments, analyzed the data and wrote the manuscript; AR performed the immunohistochemistry; EF performed the Morris water maze experiments; RV performed the statistical evaluation; CB contributed to the design of the experiments; AC contributed to the design of the experiments, performed the ELISA assays, and edited the manuscript; SO designed the experiments, analyzed the data and wrote the manuscript. All authors read and approved the final manuscript.
