# Supplementary material for: Temporal and regional progression of Alzheimer’s disease‐like pathology in 3xTg‐AD mice
Source: Aging Cell. 2018 Nov 28;18(1):e12873. doi: 10.1111/acel.12873 (PMC6351836; doi:10.1111/acel.12873)
Supplement: Supplementary file 1 [file ACEL-18-e12873-s001.pdf]

SUPPLEMENTARY MATERIAL

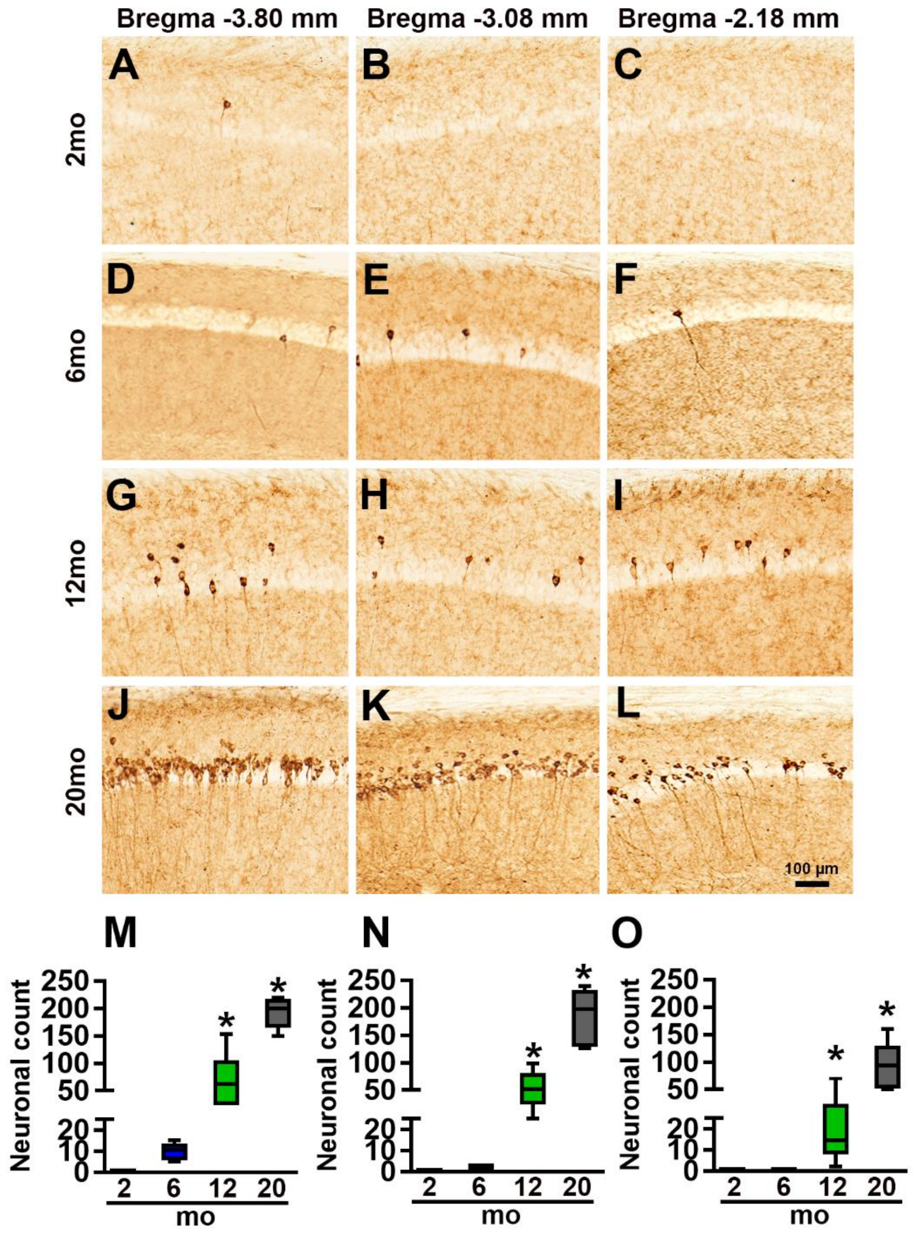

Supplemental Figure 1. Age-dependent progression of AT8-immunoreactivity in hippocampi of 3xTg-AD mice. (A-L) Representative microphotographs of hippocampal sections from 2-, 6-, 12-, and 20-month-old 3xTg-AD mice stained with an AT8 antibody, which recognizes tau phosphorylated at Ser202/Thr205. Brain sections were selected at -3.80 mm, -3.08 mm, and -2.18 mm posterior to bregma (n = 6 mice /age group). **(M-O)** Quantitative analysis of the anti-

AT8 immunoreactivity by one-way ANOVA followed by a Bonferroni's multiple-comparison test for the caudal ($p < 0.0001$, $F_{(3, 20)} = 59.57$), medial ($p < 0.0001$, $F_{(3, 20)} = 52.05$), and rostral hippocampus ($p < 0.0001$, $F_{(3,20)} = 16.01$). *Post hoc* analysis indicated that AT8 immunoreactivity in the caudal hippocampus (Bregma -3.80 mm) was significantly increased between 6 and 12 months and between 12- and 20-month-old mice 3xTg-AD ($p = 0.003$ and $p < 0.0001$, respectively). In the medial (Bregma -3.08) and rostral (Bregma -2.18) hippocampus, AT8 immunoreactivity was significantly different between 6- and 12-month-old mice ($p = 0.04$ and $p = 0.05$, respectively) and between 12- and 20-month-old mice ($p = 0.0001$ and $p < 0.0001$, respectively). Asterisks indicate differences within all the groups. Error bars represent mean ± SEM.

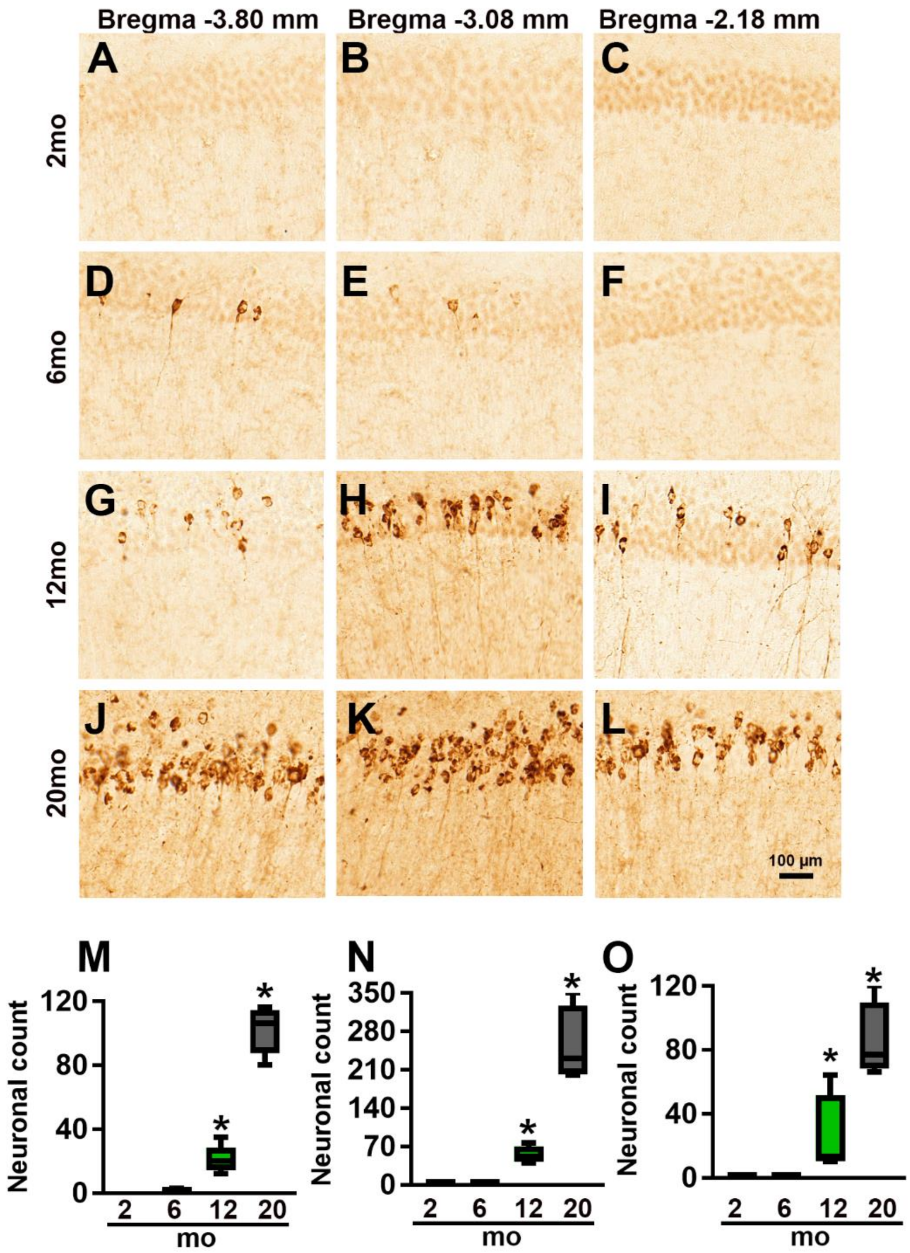

Supplemental Figure 2. Age-dependent progression of AT100-immunoreactivity in hippocampi of 3xTg-AD mice. (A-L) Representative microphotographs of hippocampal sections from 2-, 6-, 12-, and 20-month-old 3xTg-AD mice stained with an anti-AT100-specific antibody, which recognizes tau phosphorylated at Thr212/Ser214. Brain sections were selected at -3.80 mm, -3.08 mm, and -2.18 mm posterior to bregma (*n* = 6 mice /age group). **(M-O)** Quantitative analysis of the anti-AT100 immunoreactivity by one-way ANOVA followed by a Bonferroni's

multiple-comparison test for the caudal ($p < 0.0001$, $F_{(3, 20)} = 145.5$), medial ($p < 0.0001$, $F_{(3, 20)} = 53.95$), and rostral hippocampus ($p < 0.0001$, $F_{(3, 20)} = 25.78$). *Post hoc* analysis indicated that AT100 immunoreactivity in the caudal hippocampus (Bregma -3.80 mm) was significantly increased between 6 and 12 months and between 12- and 20-month-old mice 3xTg-AD (p = 0.023 and p < 0.0001, respectively). In the medial (Bregma -3.08) and rostral (Bregma -2.18) hippocampus, AT100 immunoreactivity was significantly different between 6- and 12-month-old mice (p = 0.002 and p = 0.003, respectively) and between 12- and 20-month-old mice ($p = 0.0002$ and $p < 0.0001$, respectively). Asterisks indicate differences within all the groups. Error bars represent mean ± SEM.

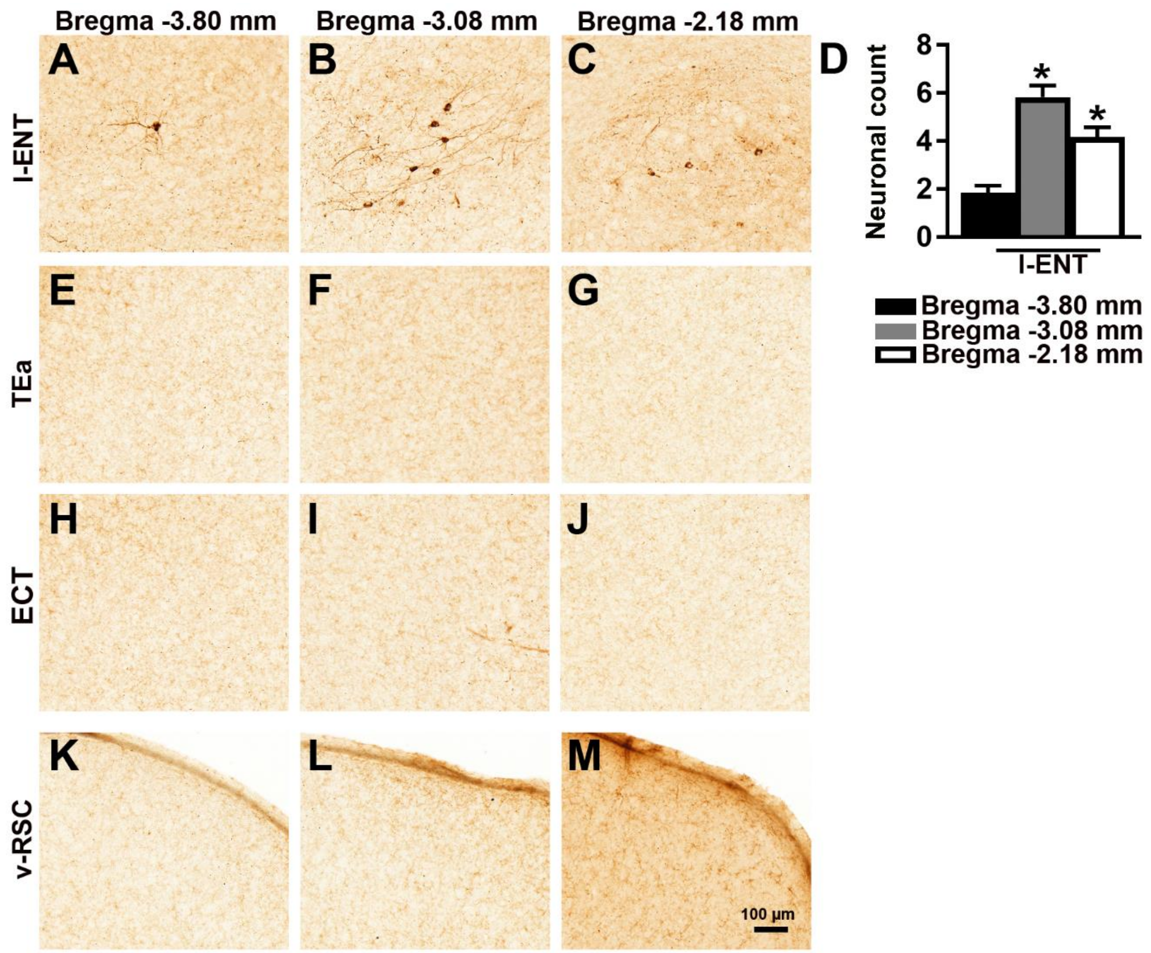

Supplemental Figure 3. Cortical pS422 immunoreactivity in 12-month-old 3xTg-AD mice. Representative microphotographs of brain sections from 20-month-old 3xTg-AD mice (n = 6) stained with a selective pS422 tau antibody. Per each brain region, sections were taken at three different rostro-caudal levels. **(A-C)** pS422 immunoreactivity in the l-ENT. **(D)** Quantitative analysis of the pS422 immunoreactivity in the l-ENT by One-way ANOVA. The number of positive neurons was higher in the medial l-ENT compared to both caudal ($p < 0.0001$) and rostral ($p = 0.0006$) l-ENT. Further, pS422 immunoreactivity was higher in the rostral compared to the caudal l-ENT ($p = 0.0025$). **(E-M)** There were no pS422-positive neurons in the other cortical regions analyzed. Asterisks indicate differences within all the groups. Error bars represent mean ± SEM.

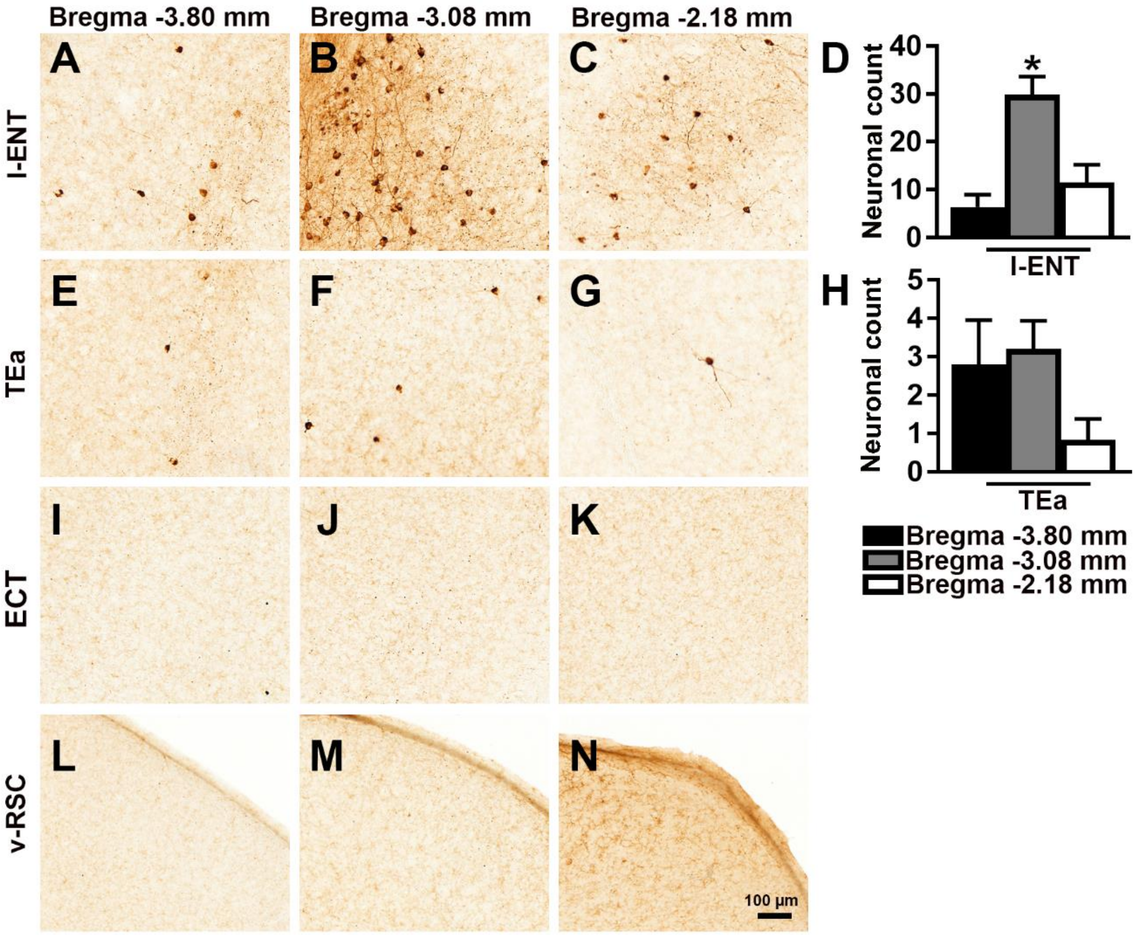

Supplemental Figure 4. Cortical tau S422 phosphorylation in 20-month-old 3xTg-AD mice. Representative microphotographs of brain sections from 20-month-old 3xTg-AD mice (n = 6/age group) stained with a selective pS422 tau antibody. Per each brain region, sections were taken at three different rostrocaudal levels. **(A-C)** pS422 immunoreactivity in the l-ENT. **(D)** Quantitative analyses of the staining indicated that the number of pS422-positive neurons was higher in the medial l-ENT compared to the caudal (p = 0.0006) and rostral l-ENT (p = 0.054). **(E-G)** pS422 immunoreactivity in the TEa. **(H)** Quantitative analyses of the staining indicated that the number of pS422-positive neurons was not significantly different among the three rostrocaudal regions of the TEa. **(i-k** and **l-n)** Lack of pS422 immunoreactivity in the ECT and v-RSC. *Asterisks indicate differences within all the groups. Error bars represent mean ± SEM.*

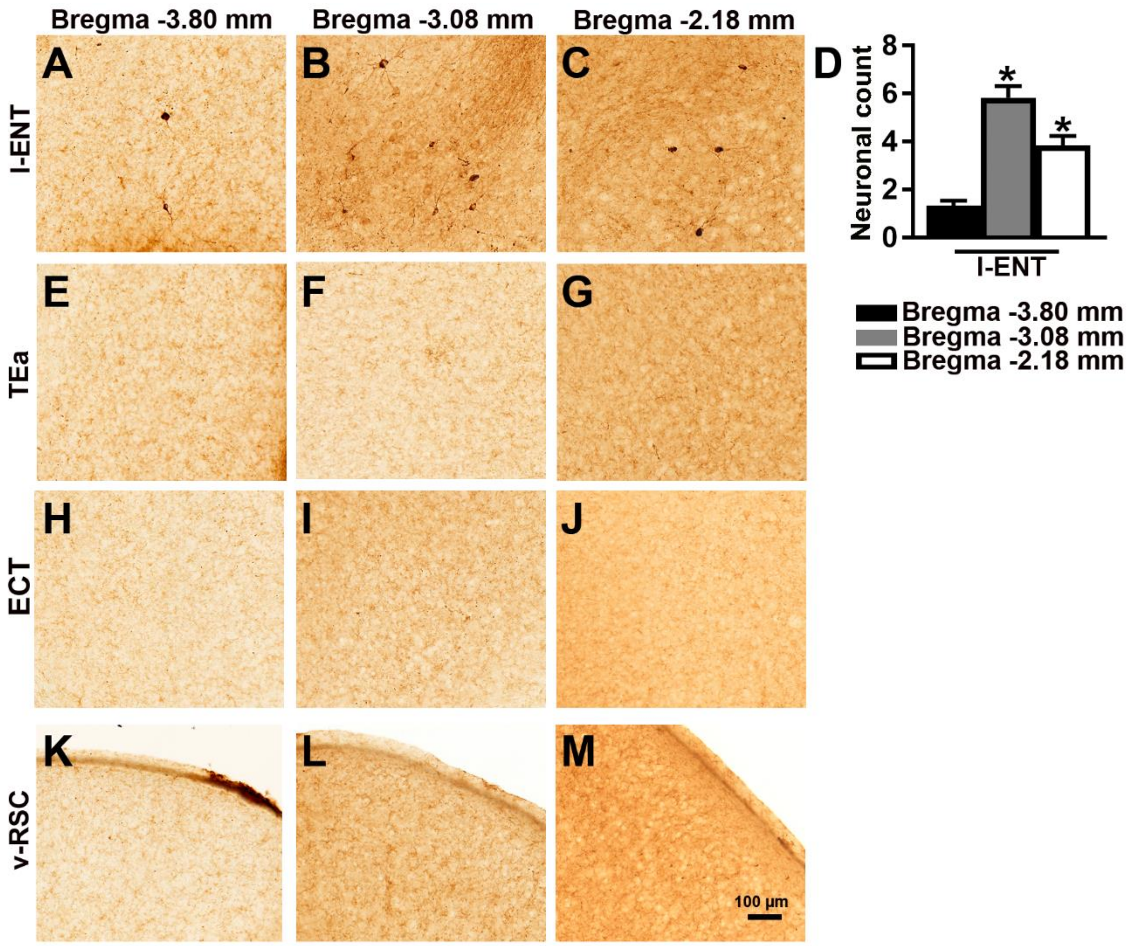

Supplemental Figure 5. Cortical AT8-immunoreactivity in 12-month-old 3xTg-AD mice. Representative microphotographs of brain sections from 12-month-old 3xTg-AD mice (n = 6) stained with AT8. Per each brain region, sections were taken at three different rostro-caudal levels. **(A-C)** AT8 immunoreactivity in the l-ENT. **(D)** Quantitative analysis of the AT8 immunoreactivity in the l-ENT by One-way ANOVA. The number of positive neurons was higher in the medial l-ENT compared to both caudal ($p < 0.0001$) and rostral ($p = 0.0061$) l-ENT. Further, AT8 immunoreactivity was higher in the rostral compared to the caudal l-ENT ($p = 0.0009$). **(E-M)** There were no AT8-positive neurons in the other cortical regions analyzed. Asterisks indicate differences within all the groups. Error bars represent mean ± SEM.

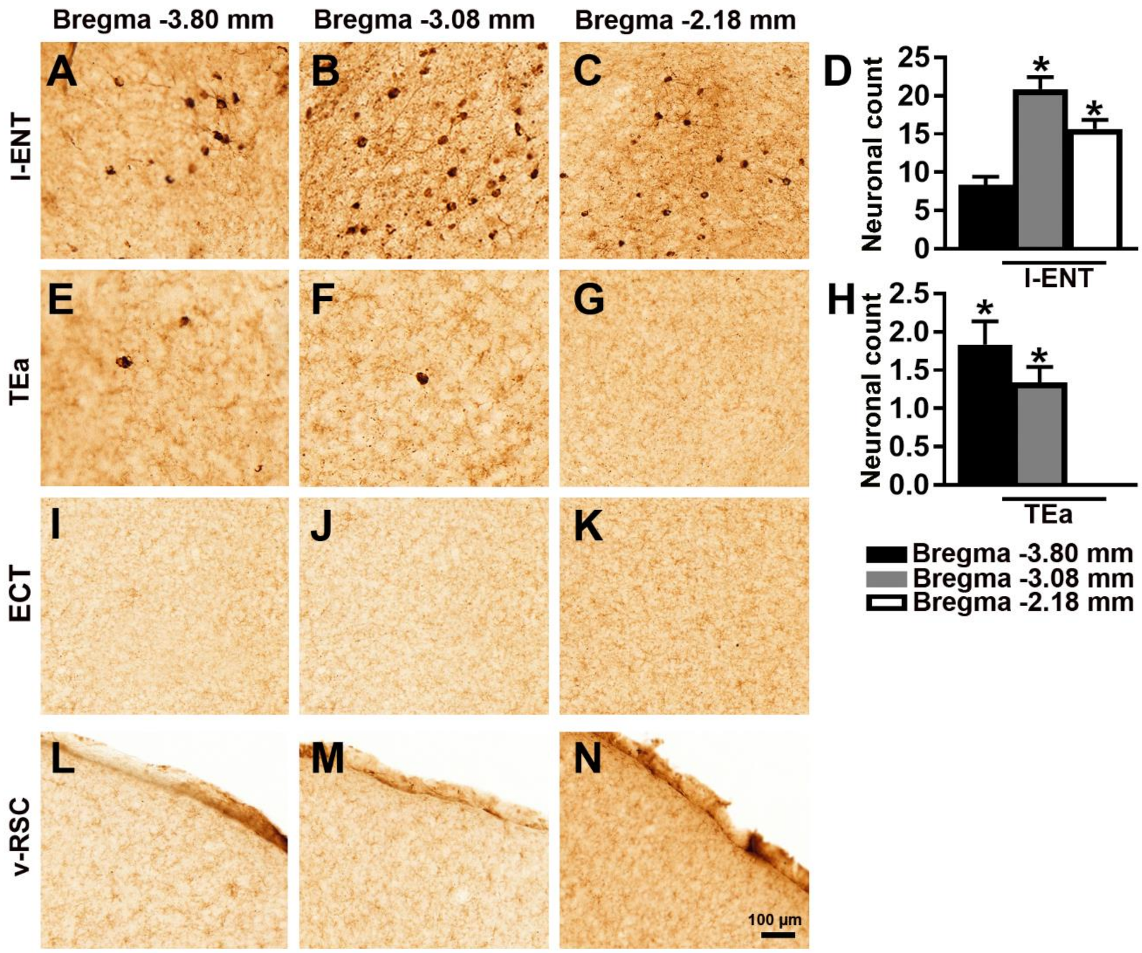

Supplemental Figure 6. Cortical AT8-immunoreactivity in 20-month-old 3xTg-AD mice. Representative microphotographs of brain sections from 20-month-old 3xTg-AD mice (n = 6) stained with AT8. Per each brain region, sections were taken at three different rostro-caudal levels. **(A-C)** AT8 immunoreactivity in the l-ENT. **(D)** Quantitative analysis of the AT8 immunoreactivity in the l-ENT. The number of positive neurons was higher in the medial l-ENT compared to both caudal ($p < 0.0001$) and rostral l-ENT ($p = 0.0436$). Also, AT8 immunoreactivity was higher in the rostral compared to the caudal l-ENT ($p = 0.0041$). **(E-G)** A limited number of AT8-positive neurons was present in the TEa. **(H)** Quantitative analysis of the AT8 immunoreactivity in the TEa shows that the number of AT8-positive neurons was significantly higher in the caudal and in the medial TEa compared to the rostral TEa ($p < 0.0001$ and $p = 0.0016$, respectively). **(I-N)** There were no AT8-positive neurons in the other cortical regions

analyzed. Asterisks indicate differences within all the groups. Error bars represent mean ± SEM.

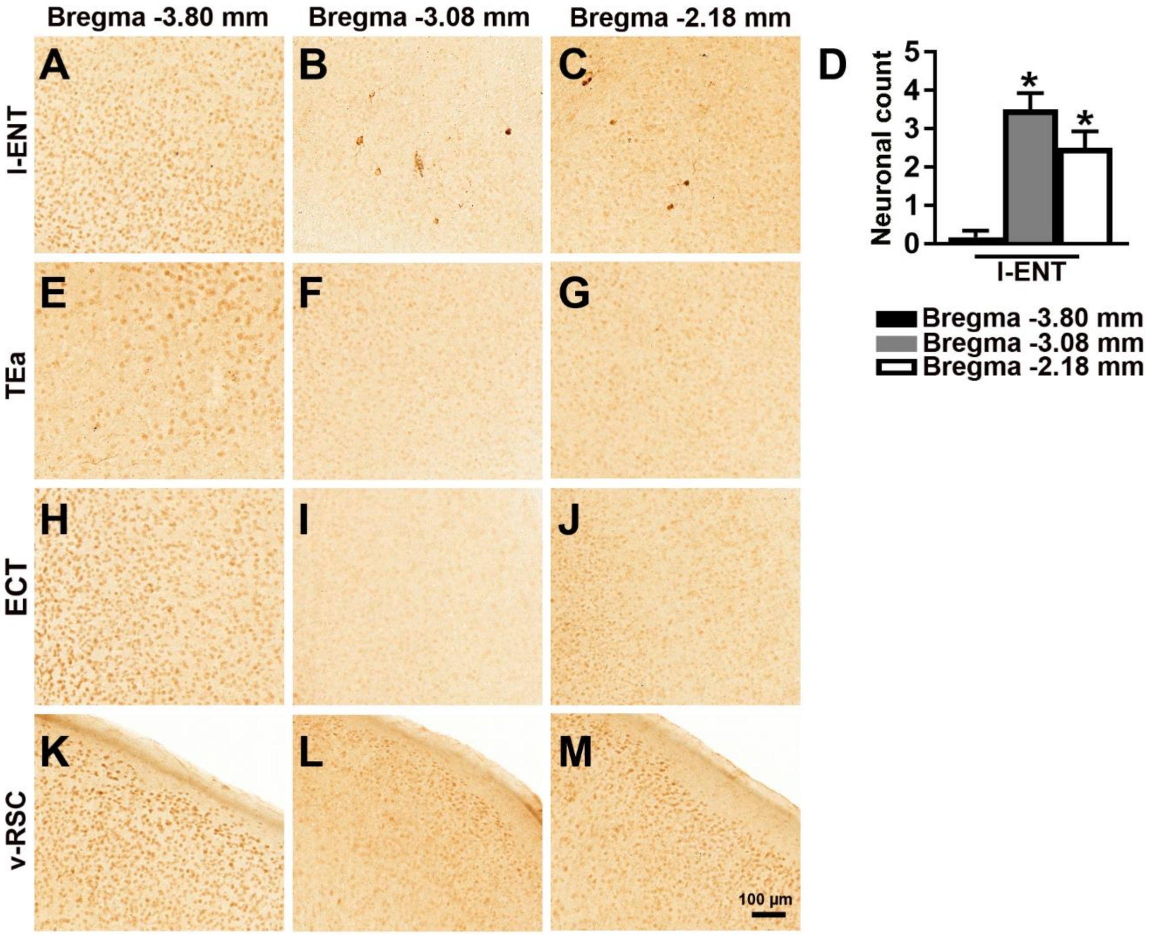

Supplemental Figure 7. Cortical Tau AT100-immunoreactivity in 12-month-old 3xTg-AD mice. Representative microphotographs of brain sections from 12-month-old 3xTg-AD mice (n = 6) stained with AT100. Per each brain region, sections were taken at three different rostro-caudal levels. **(A-C)** AT100 immunoreactivity in the l-ENT shows a limited number of AT100-positive neurons. **(D)** Quantitative analysis of the AT100 immunoreactivity in the I-ENT by One-way ANOVA. The number of positive neurons was higher in the medial l-ENT compared to the caudal ($p < 0.0001$) and rostral ($p = 0.0011$) l-ENT. **(E-M)** There were no AT100-positive neurons in the other cortical regions analyzed. Asterisks indicate differences within all the groups. Error bars represent mean ± SEM.

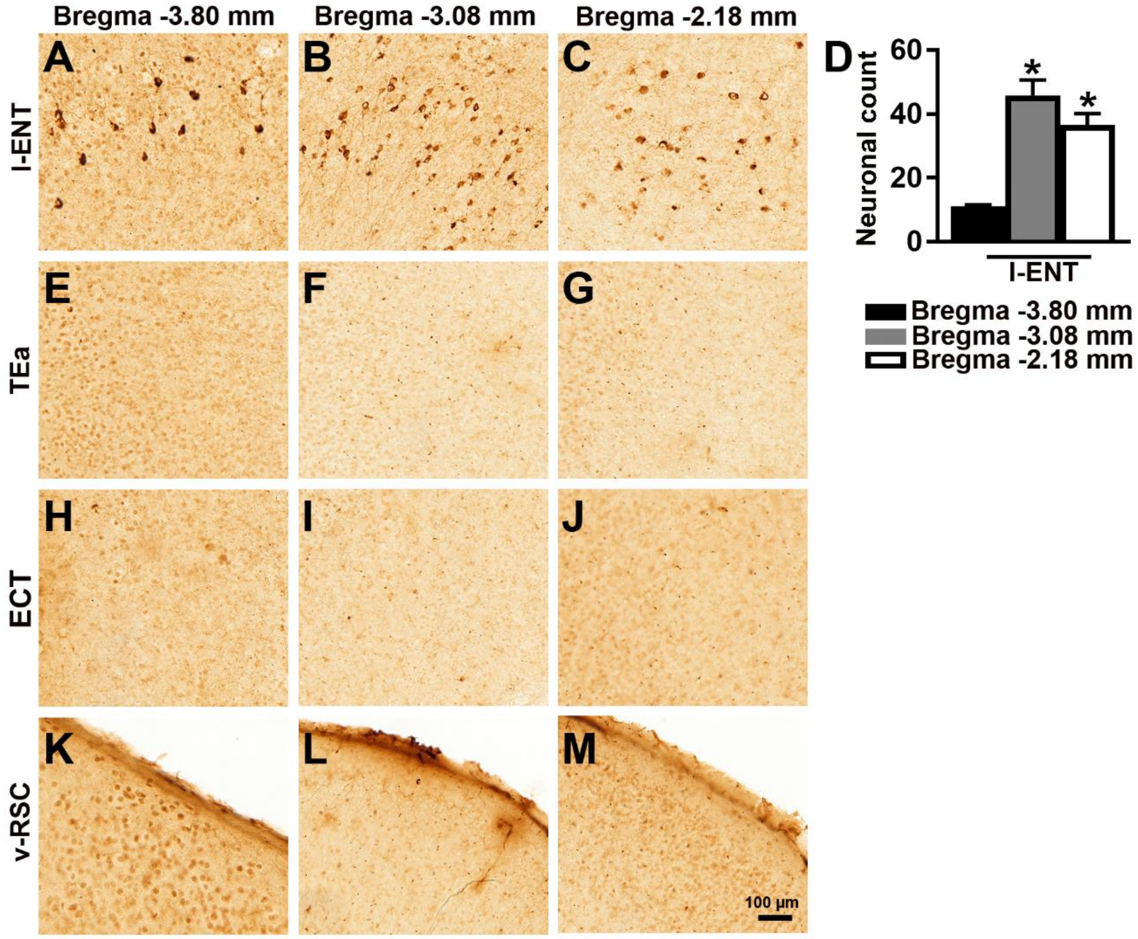

Supplemental Figure 8. Cortical Tau AT100-immunoreactivity in 20-month-old 3xTg-AD mice. Representative microphotographs of brain sections from 20-month-old 3xTg-AD mice (n = 6) stained with AT100. Per each brain region, sections were taken at three different rostro-caudal levels. **(A-C)** AT100 immunoreactivity in the l-ENT. **(D)** Quantitative analysis of the AT100 immunoreactivity in the l-ENT by One-way ANOVA. The number of positive neurons was higher in the medial l-ENT compared to the caudal ($p < 0.0001$) and rostral ($p = 0.0004$) l-ENT. **(E-M)** There were no AT100-positive neurons in the other cortical regions analyzed. Asterisks indicate differences within all the groups. Error bars represent mean ± SEM.

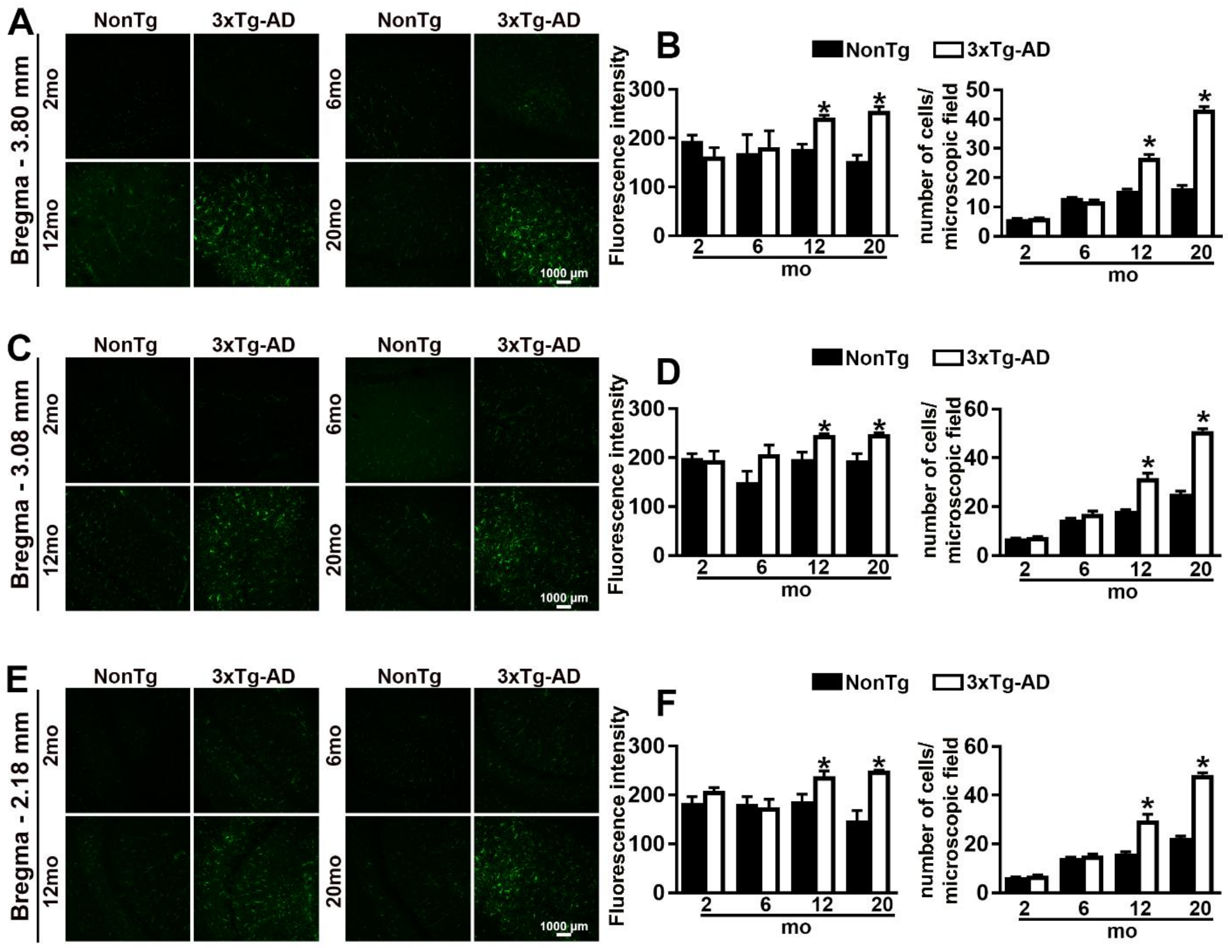

Supplemental Figure 9. Age-dependent astrogliosis in 3xTg-AD mice. Representative confocal microphotographs of 3xTg-AD and NonTg CA1 sections stained with a GFAP antibody (n = 6/genotype/age group). Per each brain region, sections were taken at three different rostrocaudal levels. **(A-B)** GFAP immunoreactivity and quantitative analyses in the caudal hippocampus. GFAP immunoreactivity and GFAP-positive cells were higher in 12- and 20-month-old 3xTg-AD mice compared to age-matched NonTg mice ($p = 0.0001$ and $p < 0.0001$, respectively). **(C-D)** GFAP immunoreactivity and quantitative analyses in the medial hippocampus. GFAP immunoreactivity and GFAP-positive cells were higher in 12- and 20-month-old 3xTg-AD mice compared to age-matched NonTg mice ($p = 0.0054$ and $p = 0.0016$, respectively). **(E-F)** GFAP immunoreactivity and quantitative analyses in the medial hippocampus. GFAP immunoreactivity and GFAP-positive cells were higher in 12- and 20-month-

old 3xTg-AD mice compared to age-matched NonTg mice ($p = 0.022$ and $p = 0.0012$, respectively). Data were analyzed by student's *t*-test. Error bars represent mean ± SEM.

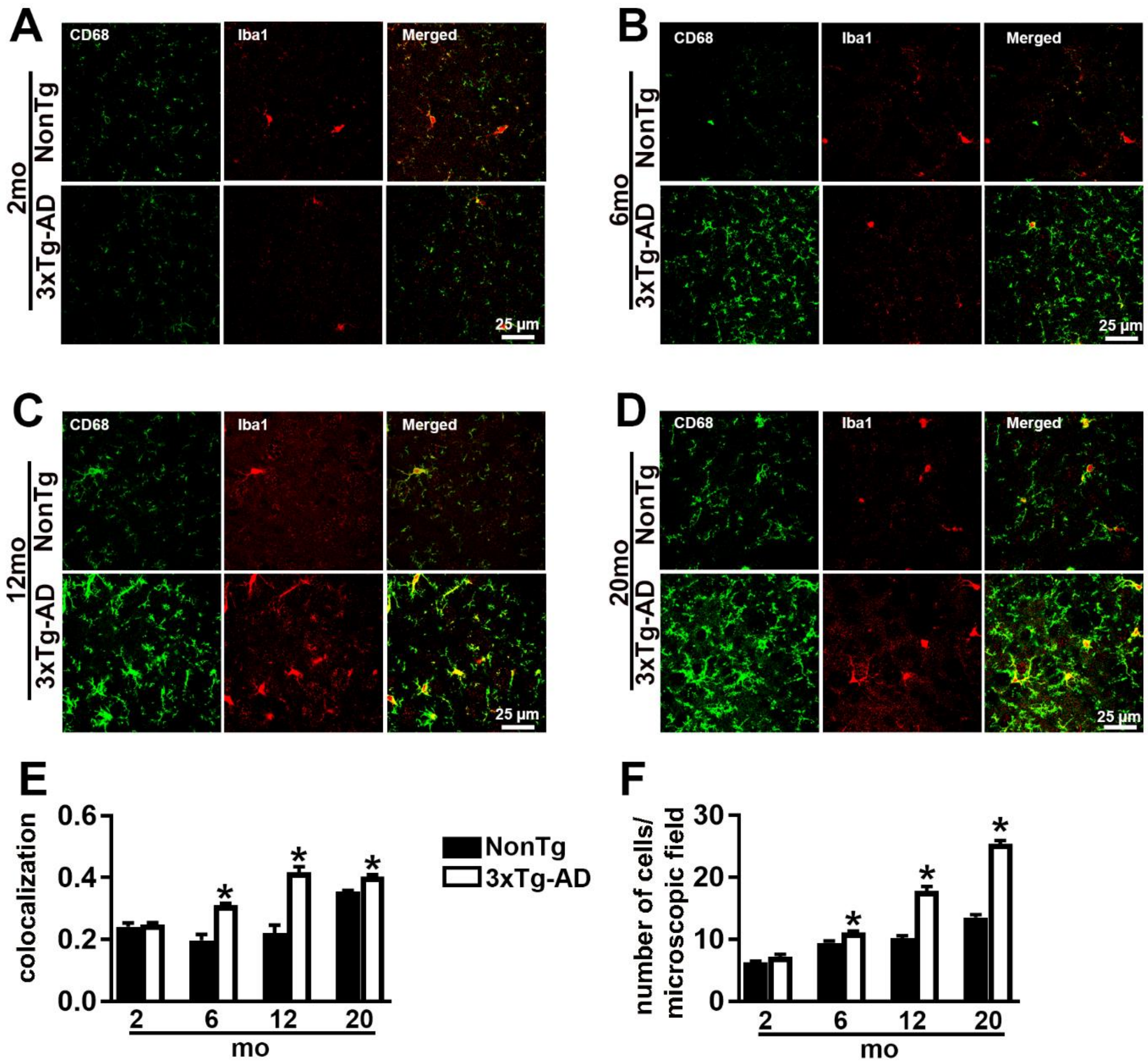

Supplemental Figure 10. Age-dependent astrogliosis in the caudal hippocampus. (A-D) Representative confocal microphotographs of CA1 sections of NonTg and 3xTg-AD mice (*n* = 6 mice/genotype/age group). Sections were stained with an anti-Iba1 and anti-CD68 antibody. **(E)** Quantitative analysis revealed that the number of colocalized pixels was significantly higher in 3xTg-AD mice than NonTg mice at 6, 12, and 20 months of age ($p < 0.0001$, $p = 0.0006$ and $p < 0.0001$, respectively). **(F)** Quantitative analysis revealed that the number of Iba1-positive cells was significantly higher in 3xTg-AD mice than NonTg mice at 6, 12, and 20 months of age. Statistical evaluation was obtained by the Pearson's correlation coefficients. Error bars represent mean ± SEM.

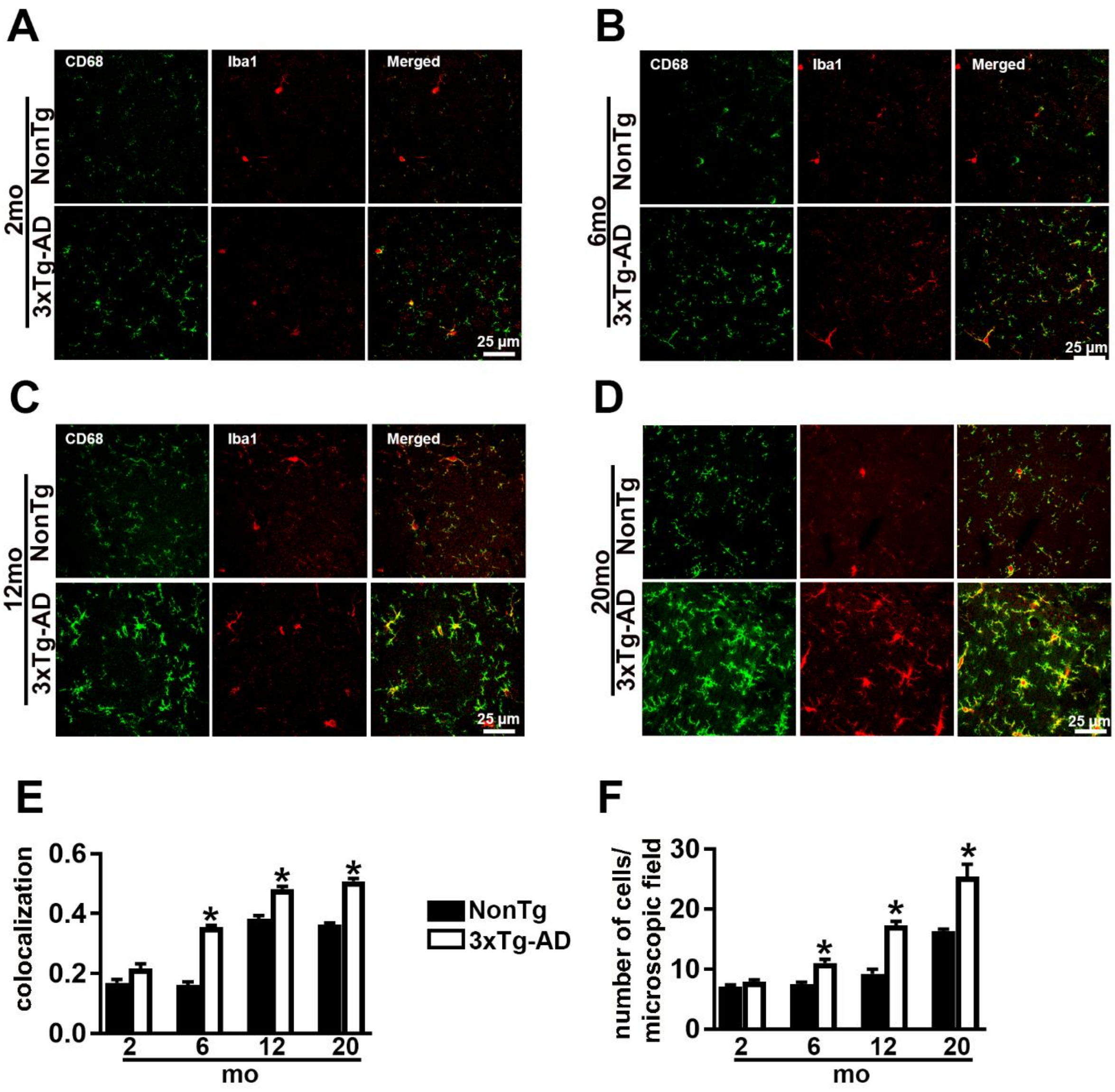

Supplemental Figure 11. Age-dependent astrogliosis in the rostral hippocampus. (A-D) Representative confocal microphotographs of CA1 sections from NonTg and 3xTg-AD mice (*n* = 6 mice/genotype/age group). Sections were stained with an anti-Iba1 and anti-CD68 antibody. **(E)** Quantitative analysis revealed that the number of colocalized pixels was significantly higher in 3xTg-AD mice than NonTg mice at 6, 12, and 20 months of age ($p < 0.0001$ for all three ages). **(F)** Quantitative analysis revealed that the number of Iba1-positive cells was significantly higher in 3xTg-AD mice than NonTg mice at 6, 12, and 20 months of age. Statistical evaluation was obtained by the Pearson's correlation coefficients. Error bars represent mean ± SEM.

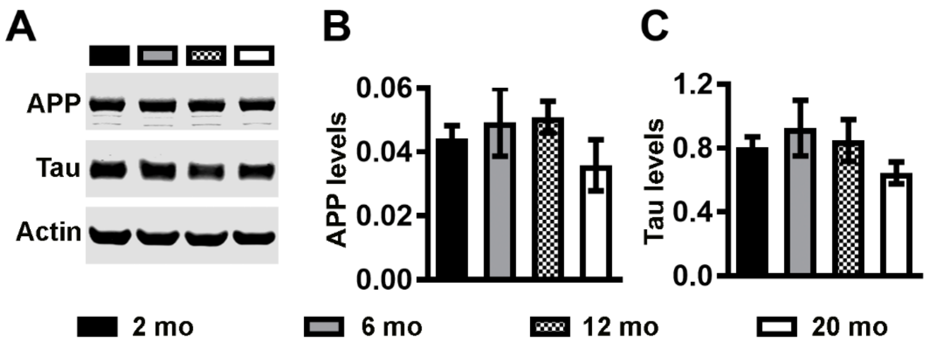

Supplemental Figure 12. Total APP and tau levels do not change as a function of age. (A) Representative western blots of proteins extracted from the hippocampi of 2-, 6-, 12-, and 20-month-old 3xTg-AD mice (n = 6/age group). To detect full-length APP, blots were probed with the 6E10 antibody; to detect full-length tau, blots were probed with the HT7 antibody. (B-C) Quantitative analyses of the blots indicated that the steady-state levels of APP and tau did not change as a function of age. Data were analyzed by one-way ANOVA. Error bars represent mean ± SEM.
